# Green upgrading of SPring-8 to produce stable, ultrabrilliant hard X-ray beams

**DOI:** 10.1107/S1600577524008348

**Published:** 2024-10-24

**Authors:** Hitoshi Tanaka, Takahiro Watanabe, Toshinori Abe, Noriyoshi Azumi, Tsuyoshi Aoki, Hideki Dewa, Takahiro Fujita, Kenji Fukami, Toru Fukui, Toru Hara, Toshihiko Hiraiwa, Kei Imamura, Takahiro Inagaki, Eito Iwai, Akihiro Kagamihata, Morihiro Kawase, Yuichiro Kida, Chikara Kondo, Hirokazu Maesaka, Tamotsu Magome, Mitsuhiro Masaki, Takemasa Masuda, Shinichi Matsubara, Sakuo Matsui, Takashi Ohshima, Masaya Oishi, Takamitsu Seike, Masazumi Shoji, Kouichi Soutome, Takashi Sugimoto, Shinji Suzuki, Minori Tajima, Shiro Takano, Kazuhiro Tamura, Takashi Tanaka, Tsutomu Taniuchi, Yukiko Taniuchi, Kazuaki Togawa, Takato Tomai, Yosuke Ueda, Hiroshi Yamaguchi, Makina Yabashi, Tetsuya Ishikawa

**Affiliations:** a RIKEN SPring-8 Center, 1-1-1 Kouto, Sayo, Hyogo679-5148, Japan; b Japan Synchrotron Radiation Research Institute (JASRI) 1-1-1 Kouto Sayo Hyogo679-5198 Japan; ESRF – The European Synchrotron, France

**Keywords:** next generation light source, accelerator, low emittance, sustainability, low-emittance storage ring

## Abstract

SPring-8-II is a major upgrade project of SPring-8 that was inaugurated in October 1997 as a third-generation synchrotron radiation light source. This upgrade project aims to achieve three goals simultaneously: achievement of excellent light source performance, refurbishment of aged systems, and significant reduction in power consumption for the entire facility. A small emittance of 50 pm rad will be achieved by (1) replacing the existing double-bend lattice structure with a five-bend achromat one, (2) lowering the stored beam energy from 8 to 6 GeV, (3) increasing the horizontal damping partition number from 1 to 1.3, and (4) enhancing horizontal radiation damping by installing damping wigglers in long straight sections. The use of short-period in-vacuum undulators allows ultrabrilliant X-rays to be provided while keeping a high-energy spectral range even at the reduced electron-beam energy of 6 GeV. To reduce power consumption, the dedicated, aged injector system has been shut down and the high-performance linear accelerator of SACLA, a compact X-ray free-electron laser (XFEL) facility, is used as the injector of the ring in a time-shared manner. This allows the simultaneous operation of XFEL experiments at SACLA and full/top-up injection of the electron beam into the ring. This paper overviews the concept of the SPring-8-II project, the system design of the light source and the details of the accelerator component design.

## Abstract 2

This paper presents an overview of a major upgrade project of SPring-8, SPring-8-II, including ultra-low-emittance accelerator design and expected performances. The paper also discusses our approach towards the next generation light source aiming at the reduction of power consumptions.

## 1. Introduction

Synchrotron radiation (SR) sources have long served as a powerful research tool in various scientific fields and industrial applications. The brilliance and stability of SR sources have seen significant improvements during the second half of the 20th century along with the evolution from the first generation to the third generation. In 2009, the advent of a free-electron laser (FEL) in the hard X-ray region (Emma *et al.*, 2010 ▸) opened up new avenues for innovative experiments, thanks to its unique characteristics of ultrafast, high peak brilliance and full transverse coherence properties. The ability of intense X-ray FEL (XFEL) pulses to capture femtosecond atomic movies, coupled with that of SR to non-destructively observe various phenomena with moderate interactions with matter, underscores the complementary nature of these two tools. Both are essential for a comprehensive understanding of phenomena of interest across multiple scales of space and time.

SR facilities, with their broad applications and established track record, are poised to make significant contributions to addressing pressing global issues, including the ambitious goal of achieving net-zero CO_2_ emissions by 2050. Aligning with these global efforts, the performance of SR sources worldwide is being upgraded to the fourth generation in a sequential manner, facilitating the transformation of production and living infrastructure into a sustainable form. This generation upgrade has been made possible by the development of multi-bend achromat (MBA) technologies (Einfeld *et al.*, 1995 ▸), which drastically reduce the emittance of the electron beam stored in a storage ring. In line with this trend, a significant upgrade of the light source performance of SPring-8 (JAERI-RIKEN SPring-8 Project Team, 1991 ▸), SPring-8-II (RIKEN-JASRI SPring-8-II Project Team, 2014 ▸; Tanaka, 2014 ▸; Watanabe & Tanaka, 2023 ▸), is under investigation.

Considering the current social conditions, the upgrade of SR facilities should not only focus on conventional performance supremacy but also on the sustainable development of the facilities themselves. The design of SPring-8-II is therefore rooted in this greener concept, aiming to achieve a significant improvement in performance and substantial savings in electric power consumption and material resources. This commitment to sustainable development reassures us of the facility’s role in the future.

## 2. Targeting performance of SPring-8-II as a fourth-generation synchrotron radiation source

**Figure 1:**
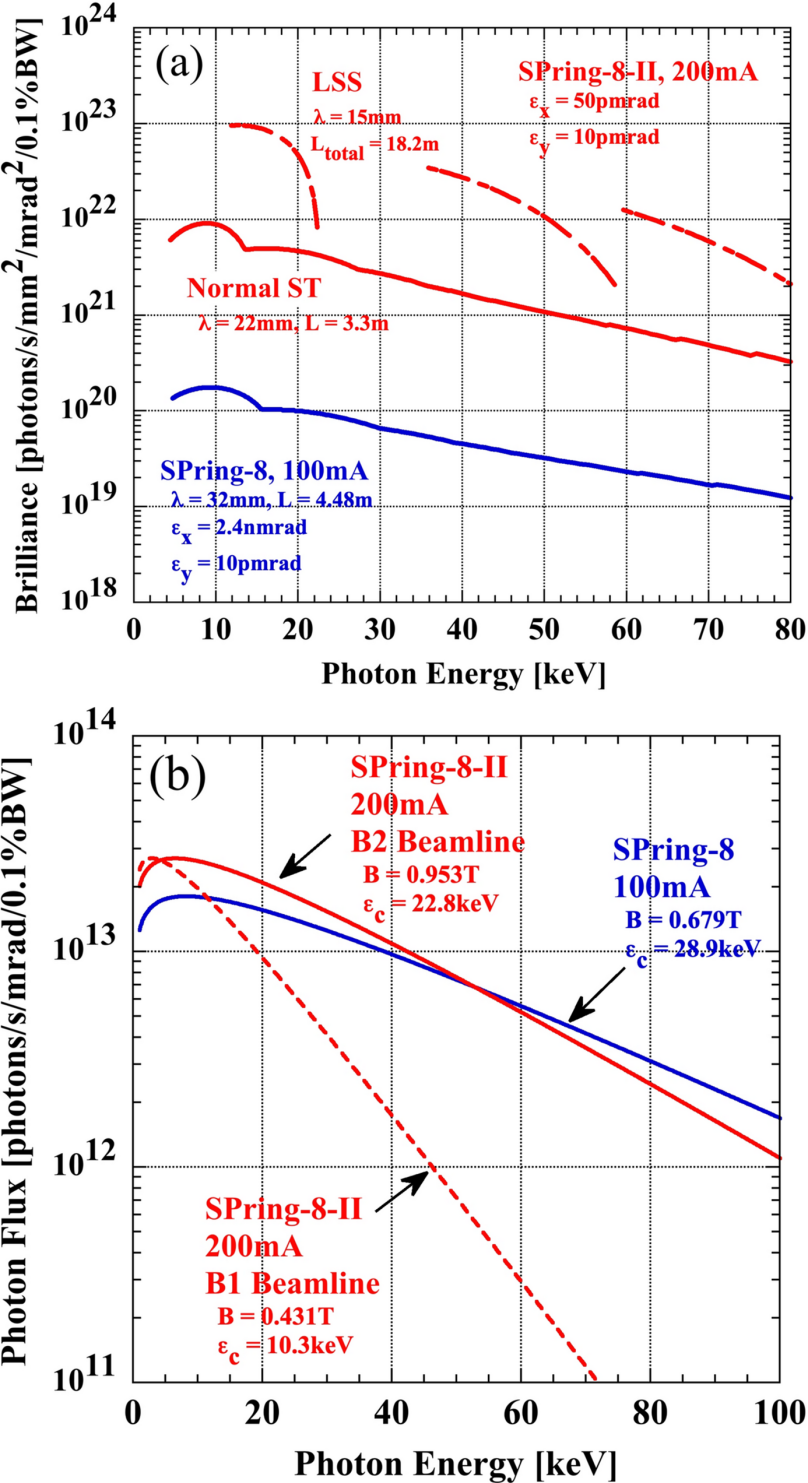
Comparison of (*a*) spectral brilliance of undulator radiation for SPring-8-II and SPring-8 up to the 13th harmonic and (*b*) photon flux of bending magnet radiation. Brilliance calculated by *SPECTRA* (Tanaka, 2021 ▸).

As highlighted in the *Introduction*[Sec sec1], our goal with SPring-8-II is not only to enhance its light source performance but also to ensure its sustainability, thereby saving electricity and resources. We have set a challenging target of a minimum emittance of 50 pm rad, which would create a considerable number of coherent portions even for high-energy X-rays, and deliver a brilliance surpassing 10^22^ photons s^−1^ mm^−2^ mrad^−2^ (0.1% bandwidth)^−1^ around 10 keV from undulators. Table 1 ▸ shows the main source parameters of SPring-8-II together with those of the current SPring-8. This target brilliance of undulator beamlines is a testament to our collective efforts, as it is approximately two orders of magnitude higher than the current SPring-8, and it underscores our commitment to pushing the boundaries of synchrotron radiation sources. Fig. 1 ▸ shows (*a*) the spectral brilliance from undulators and (*b*) photon flux from dipole magnets available with SPring-8-II compared with those with the current SPring-8. In our design, radiation not only from undulators but also from bending magnets is considered, supposing that we will also deliver radiation to existing bending magnet beamlines. For so-called B2 beamlines where radiation from the second bending magnets of the current double-bend lattice is delivered, almost the same spectral range of radiation will be covered for SPring-8-II by the newly designed lattice as shown below.

In the design of the SPring-8-II source with the above target performance, our operational experience at SPring-8 Ångström Compact free-electron LAser (SACLA) (Ishikawa *et al.*, 2012 ▸) has played a critical role in the functioning of an ultralow emittance ring as a practical light source. The key issue is to ensure sufficient stability and reproducibility of a small, well collimated photon beam for individual beamlines. Based on these considerations, we defined the target criteria for SPring-8-II, which are summarized as follows:

(*a*) Transparent tuning of the individual insertion devices (IDs). To perform precision experiments at multiple beamlines simultaneously, the tuning of the gap (and phase) of an ID at a given beamline should not affect the condition of light sources at other beamlines. To achieve this criterion for transparent tuning, each ID should not introduce any orbit distortion while changing its gap. Moreover, consideration should be given to reducing the fluctuations in emittance that can occur with the change in radiation power caused by the gap change.

(*b*) Ultimate stability of the electron beam. Advanced X-ray focusing devices have generated small X-ray spots in the third-generation SR for micro- and nano-analysis. Conventionally, a small aperture that acts as a virtual source is inserted into a beamline. However, the fourth-generation SR with reduced source size can eliminate the aperture, allowing a drastic enhancement of the photon flux in the nano beam. In this case, the stability of the electron beam must be ensured to avoid blurring of the focused beam. Although we have already achieved this criterion at SACLA, where the direct imaging of the light source is routinely utilized at the beamlines (Yumoto *et al.*, 2013 ▸; Mimura *et al.*, 2014 ▸; Yamada *et al.*, 2024 ▸), serious consideration is required for SPring-8-II. In particular, the ripple of the electric current of a power supply for an electromagnet and the vibration of a vacuum chamber have been major sources of electron orbit fluctuations in the present SPring-8, and they should be sufficiently suppressed in SPring-8-II.

(*c*) Transparent top-up beam injection. During top-up beam injection, the influence on the experiments performed at the beamlines should be minimized. To achieve this ‘transparent’ top-up injection condition, the spatial deviation of the stored beams should be suppressed to as small as tens of micrometres (1σ), which is less than one-third of the horizontal beam size.

(*d*) Optical axis monitoring for electron-beam orbit correction. At SACLA, a non-destructive, in-line optical monitoring system that is capable of monitoring the optical axis during the experiment, and quick correction system of the electron-beam orbit, have been developed and routinely operated (Tono *et al.*, 2011 ▸). A similar system is highly useful and should be developed for maintaining high stability and reproducibility at the beamlines of SPring-8-II.

## 3. The design concept of the SPring-8-II accelerator system

The SPring-8-II project is a major refurbishment of SPring-8 light source that has been operated for more than a quarter of a century. The boundary conditions imposed are thus similar to those of other upgrade projects (Dimper *et al.*, 2014 ▸; Braun *et al.*, 2021 ▸; APS, 2019 ▸), as follows:

(1) Reuse of the existing ring tunnel.

(2) Minimization of a blackout period to approximately one year.

(3) Utilization of all ID beamlines with preservation of the photon beam axes.

(4) Utilization of most bending magnet (BM) beamlines.

(5) Preservation of an available spectral range with a focus on higher photon energy.

**Figure 2:**
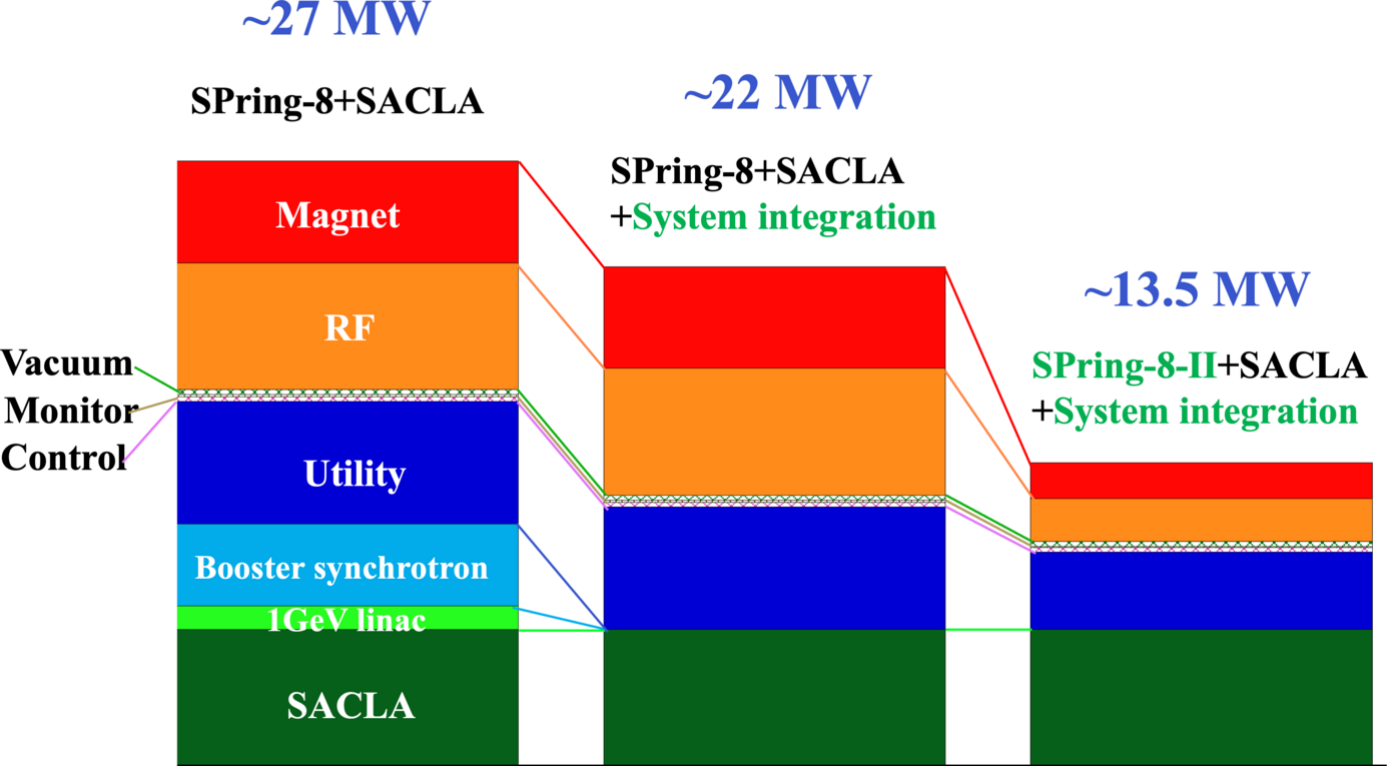
Power consumption at three stages: previous, present and future accelerator complexes at the SPring-8 site (from left to right).

The design concept comprises four pillars. We plan to achieve a design target of 50 pm rad while simultaneously reducing power consumption and resource usage under the above constraints.

(*a*) Adoption of an MBA lattice with damping wigglers (DWs). The MBA lattice was adopted to access a design emittance of 50 pm rad with the assistance of DWs installed in 30 m-long straight sections (LSSs), originally constructed in SPring-8. In order to accommodate the discrete photon absorbers in a compact vacuum chamber, a five-bend achromat (5BA) lattice was chosen. Longitudinal and transverse gradient bending magnets reduce the emittance to about 111 pm rad by suppressing the radiation excitation and increasing the horizontal damping partition number. In our design, the emittance can be reduced in stages from 111 to 50 pm rad by increasing the number of DWs in LSSs.

(*b*) Reduction of the beam energy by introducing short-period in-vacuum undulators (Yamamoto *et al.*, 1992 ▸; Hara *et al.*, 1998 ▸). Over the past 30 years, the advent of undulator technology has drastically reduced the lower limit of an undulator period down to ∼20 mm and even smaller. The introduction of this advanced technology has allowed us to reduce the stored beam energy from 8 to 6 GeV, while maintaining the available spectral range. Also, this energy reduction helps reduce power consumption in accelerator components.

(*c*) Replacement of a part of the electromagnets with permanent magnets: We replace bending electromagnets used at a constant magnetic field with permanent-type bending magnets (Watanabe *et al.*, 2017 ▸). DC septum electromagnets, which consume a large amount of power, are also replaced with permanent-type DC septum magnets (Taniuchi *et al.*, 2020 ▸). These replacements significantly contribute to power savings.

(*d*) Time-sharing of the SACLA linac as a ring injector. A beam injection scheme is one of the most challenging issues in designing an extremely low emittance storage ring. Such a storage ring naturally has a small dynamic aperture, making it difficult to apply a simple nonlinear kicker scheme that cannot sufficiently reduce the injection beam amplitude (Atkinson *et al.*, 2011 ▸). The on-axis beam injection method ideally allows zero injection amplitude (Emery & Borland, 2003 ▸), but the uncertainty in the reliability of the high-precision, ultrafast kickers in series and the complex beam abort process poses significant risks to stable and reliable beam operation. We therefore decided to adopt an advanced off-axis beam injection scheme (Takano *et al.*, 2019 ▸), which takes full advantage of the ability to accommodate short-pulse low-emittance injection beams from the SACLA linear accelerator. This scheme can achieve sufficient beam injection performance (*i.e.* both small injection beam amplitude and small stored beam oscillation). In addition, we can significantly reduce the power consumption by 5 MW by eliminating the operation of a dedicated injector system consisting of a 1 GeV linac and an 8 GeV booster synchrotron (Hara, 2022 ▸; Tanaka, Inagaki *et al.*, 2023 ▸). Fig. 2 ▸ shows the reduction in power consumption of SPring-8-II compared with those of the previous and current SPring-8.

## 4. SPring-8-II accelerator component systems

### 4.1. Beam injector

#### 4.1.1. Timesharing of SACLA linear accelerator as a ring injector

**Figure 3:**
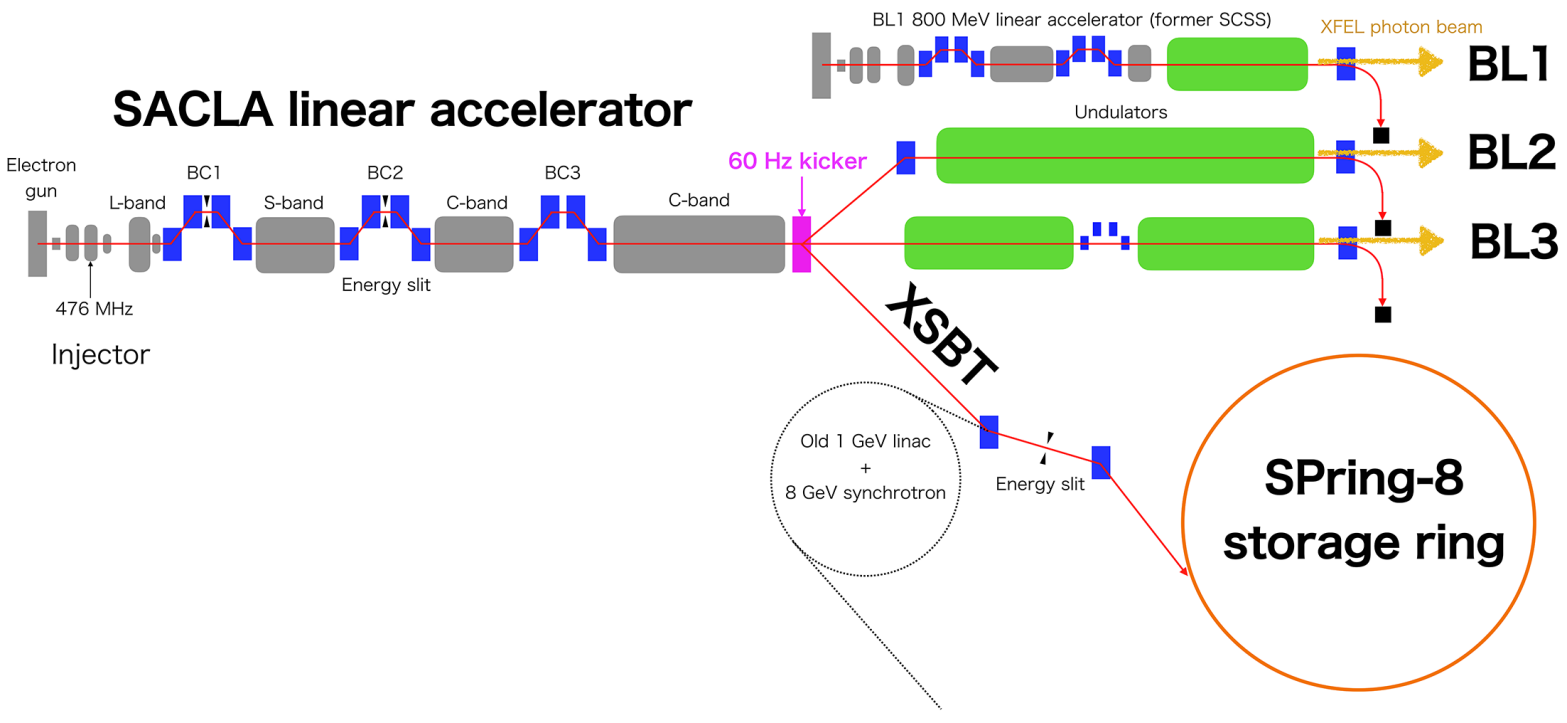
Layout of SACLA.

**Figure 4:**
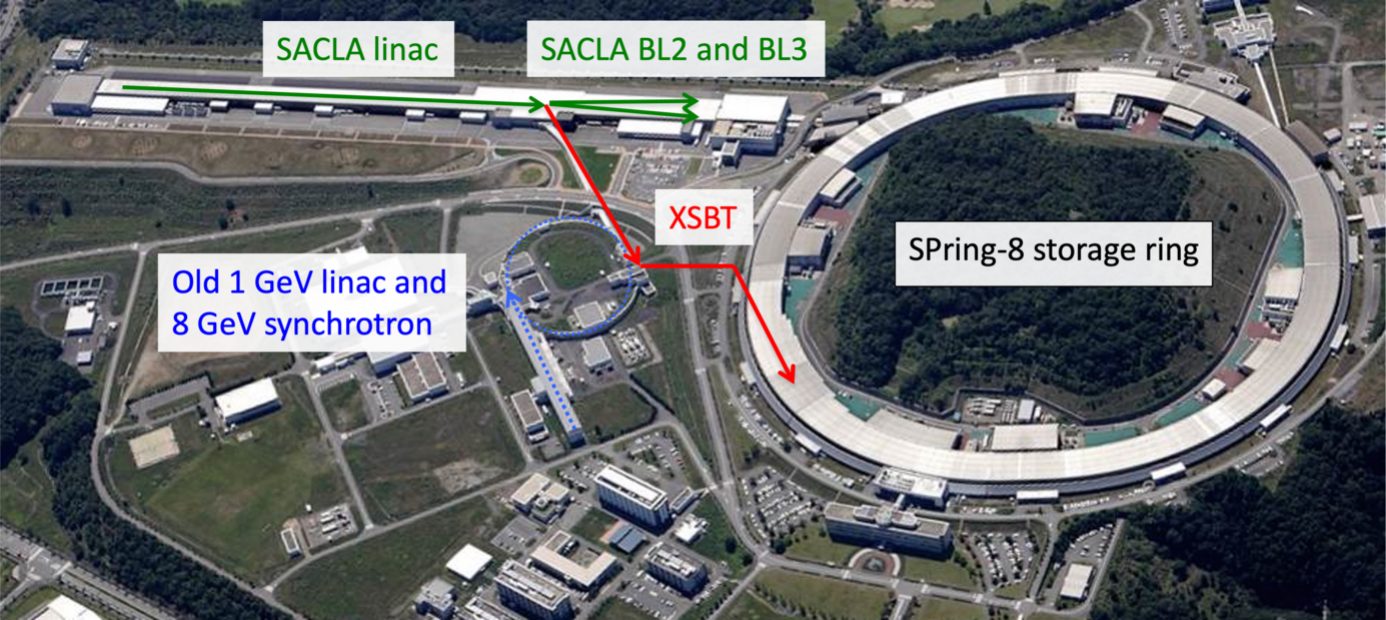
Panoramic view of the SPring-8 campus.

Fig. 3 ▸ shows a layout of the SACLA facility (Ishikawa *et al.*, 2012 ▸). Three FEL beamlines are installed in the SACLA undulator hall and experimental hall: a soft X-ray beamline BL1 and hard X-ray beamlines BL2 and BL3. BL1 is driven by a dedicated 800 MeV linear accelerator, which was originally constructed as a test bench of SACLA, the SCSS test accelerator (Owada *et al.*, 2018 ▸). BL2 and BL3 share the 60 Hz electron beam from the SACLA linear accelerator using a kicker magnet installed at the end of the accelerator (Hara *et al.*, 2016 ▸; Kondo *et al.*, 2018 ▸). For the beam injection to the SPring-8 storage ring, the electron beam is deflected opposite to BL2 (Hara *et al.*, 2021 ▸). Then the electron beam is transported through the XFEL to storage ring beam transport (XSBT) and injected into the storage ring. A panoramic view of the SPring-8 campus is shown in Fig. 4 ▸. The first half of XSBT, from the SACLA linear accelerator to the exit of a former 8 GeV injector synchrotron, was newly designed and constructed along with the SACLA facility, while the second half is a reuse of an old existing beam transport line. A double-bend achromat (DBA) is chosen as beam optics for the first half to avoid beam emittance growth but the second half of XSBT retains the original FODO lattice with large energy dispersion functions. Although the emittance is degraded by the second half of XSBT, it still meets the requirements for beam injection as we will discuss later.

During the XFEL user operation, the electron beam energies are frequently changed to adapt the X-ray wavelengths to the requirements of the users at the XFEL beamlines. On the other hand, the beam injection to SPring-8 requires an electron beam with a fixed energy. To accommodate both the XFEL operation and the beam injection, the energy of each electron bunch is changed according to the beam route (Hara *et al.*, 2013 ▸). In order to control the electron bunch energy, a multi-energy operation scheme was developed, in which the number of C-band accelerating structures used for acceleration and RF phases is changed from bunch to bunch.

#### 4.1.2. Pulse-by-pulse beam route control system

To control the beam route among BL2, BL3 and XSBT, we use a software process that switches the beam route bunch-by-bunch according to a route pattern table. The process is executed on a CPU module of MicroTCA.4 standard equipped with the timing synchronization system (Maesaka *et al.*, 2019*a* ▸), which distributes the route information to the RF acceleration units and the kicker magnet via a reflective memory network. Each subsystem has a lookup table that contains set values of accelerator components for each route, such as the phase angle of an RF acceleration unit or the excitation current of a magnet. The parameters of each subsystem are changed bunch-by-bunch by a local CPU module or a PLC.

To simplify the beam route switching control, we change the beam route pattern every second. Some 60-shot beam route pattern tables are prepared and the beam route and parameter switching system changes the table every second (Maesaka *et al.*, 2021 ▸). The same route pattern table is repeatedly used when the beam is delivered only to the XFEL beamlines or the beam is used only for the injection into the storage ring to fill up from zero current. For top-up beam injection, however, one bunch should be provided to the storage ring upon request during the usual XFEL operation. When the storage ring requests a top-up beam injection, an injection request including the target bucket address is sent to the switching system, which inserts the route pattern table having a beam injection shot. In this way, a beam for top-up injection is delivered to the storage ring on demand within 2 s.

#### 4.1.3. Timing system

An injected electron beam from the SACLA linac to the SPring-8-II storage ring must be synchronized to the desired RF bucket timing of the storage ring. The timing jitter is required to be less than 10 ps r.m.s. to suppress the synchrotron oscillation of the injected beam. Since the reference RF signal of SACLA is independent of that of the storage ring, an additional synchronization mechanism is required for the beam injection. Therefore, we have developed a new timing synchronization system for the linac of SACLA (Ohshima *et al.*, 2019 ▸). This system consists of a MicroTCA.4-based digitizer and a rear transition module (RTM) for synchronization. The input signals for the RTM are the commercial AC 60 Hz, the linac reference RF signal of 238 MHz, and the storage ring reference RF signal of 509 MHz. This module generates a master trigger that governs linac timing-dependent subunits. The electron beam is ejected 15.5 ms after this trigger. The first step to synchronize is a rough adjustment of the timing of the linac master trigger to the storage ring bucket timing signal. The master trigger signal is generated by re-clocking the zero-cross timing of the AC 60 Hz with the storage ring revolution signal of 209 kHz, and the linac reference RF signal of 238 MHz. The 209 kHz revolution signal is generated by counting the 509 MHz signal in the RTM. The next step is fine-tuning by applying FM control to the linac master oscillator. The timing deviation between the storage ring revolution signal and the linac reference RF signal is measured by detecting the phase difference between the two RF signals. We feed one-third of the 238 MHz signal (79.3 MHz) to an analog-to-digital converter (ADC) whose clock frequency is 406 times 209 kHz (84.9 MHz). The data from the ADC has a 5.5 MHz sine wave signal form and it is compared with the 5.5 MHz numerically controlled oscillator (NCO). The NCO is reset by the master trigger signal so that the phase difference between the two sine waves is proportional to the timing difference between the linac and the injection bucket. By using this phase signal, FM feedback control is applied to the master oscillator of the linac. These timing modifications are applied only to the storage ring beam injection shot and finished within 16.6 ms to minimize the perturbation to the XFEL shots. We achieved a beam arrival timing jitter of approximately 3 ps r.m.s. to the bucket timing. This system has been working with the present SPring-8, and it can be extended to the case of the upgraded ring, where the storage ring RF frequency is changed from 508.58 MHz to 508.764 MHz.

#### 4.1.4. Low-emittance beam injection to present SPring-8 storage ring

**Figure 5:**
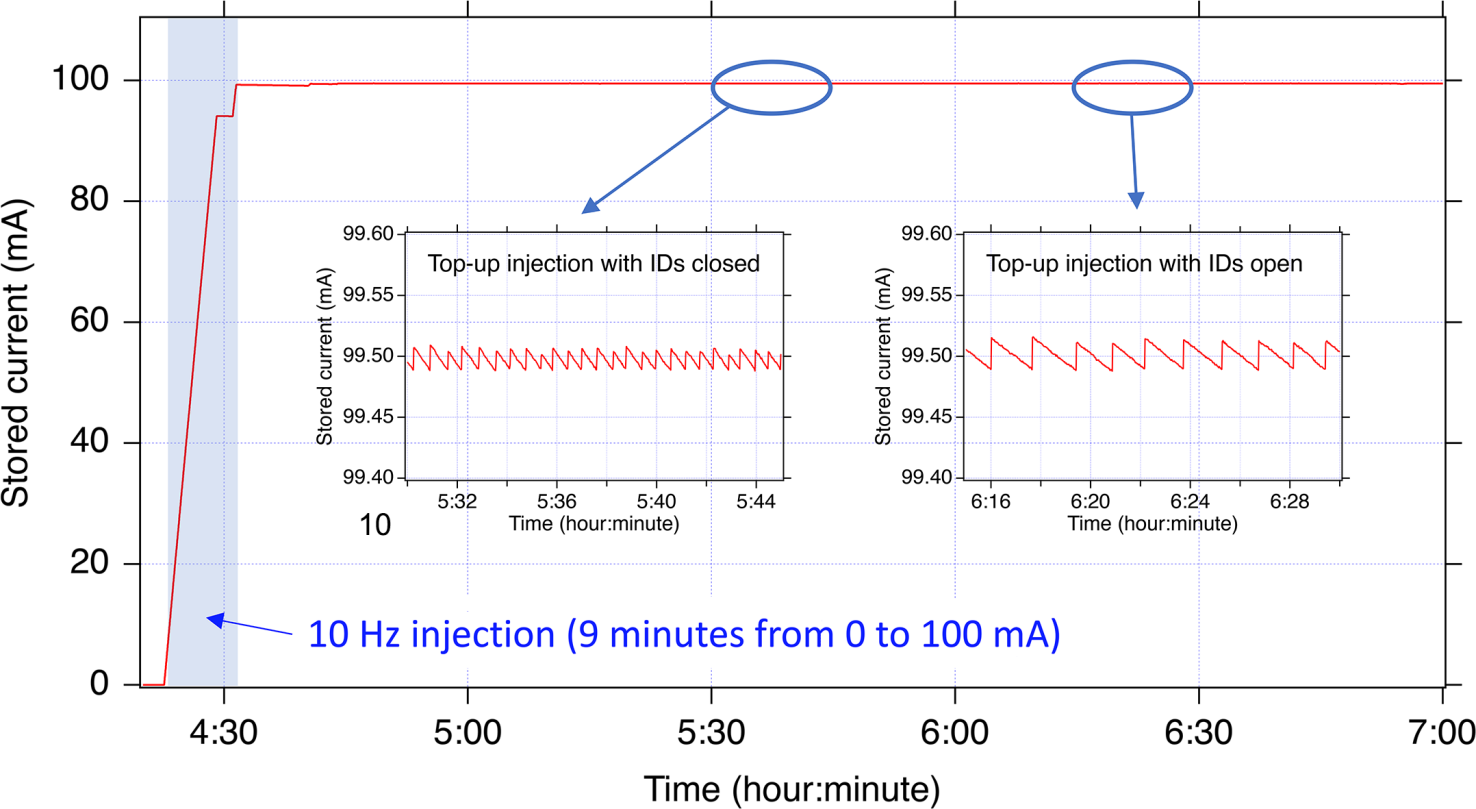
Stored current of SPring-8 during the beam injection from SACLA.

Since 2020, SACLA has been used as a full-energy injector of the present SPring-8 storage ring, and the old injector complex, consisting of a 1 GeV linear accelerator and an 8 GeV booster synchrotron, has been shut down. Fig. 5 ▸ shows the accumulated current of the SPring-8 storage ring during the beam injection. The injection efficiency is close to 100%. The beam injection from the old 8 GeV synchrotron was performed at 1 Hz. However, the injection rate was increased to 10 Hz considering the relatively small electron bunch charge of approximately 150 pC from SACLA. The time required for injection to attain the nominal stored current of 100 mA from 0 mA is about 10 min, which is half the time compared with that of the previous injector. After filling up to 100 mA, the stored current is maintained constant through top-up injection as shown in Fig. 5 ▸. The frequency of top-up injections is two to three times per minute depending on the magnetic gaps of the in-vacuum undulators and a filling pattern of the electron bunches. During the accumulation injection from 0 mA, which is typically performed once a week to change the filling pattern, XFEL user operation is suspended. The top-up injection, on the other hand, is conducted in parallel with XFEL user operation upon an injection request from SPring-8. The XFEL users need to distinguish the pulses omitted due to the top-up injection and ensure the integrity of their experimental data. In SACLA, a tag number is assigned to every electron bunch, and those not provided properly to user experiments, due to RF malfunction or the beam injection, are recorded in a database. This allows the user to identify missing or abnormal pulses due to the top-up injection and other abnormal phenomena.

**Figure 6:**
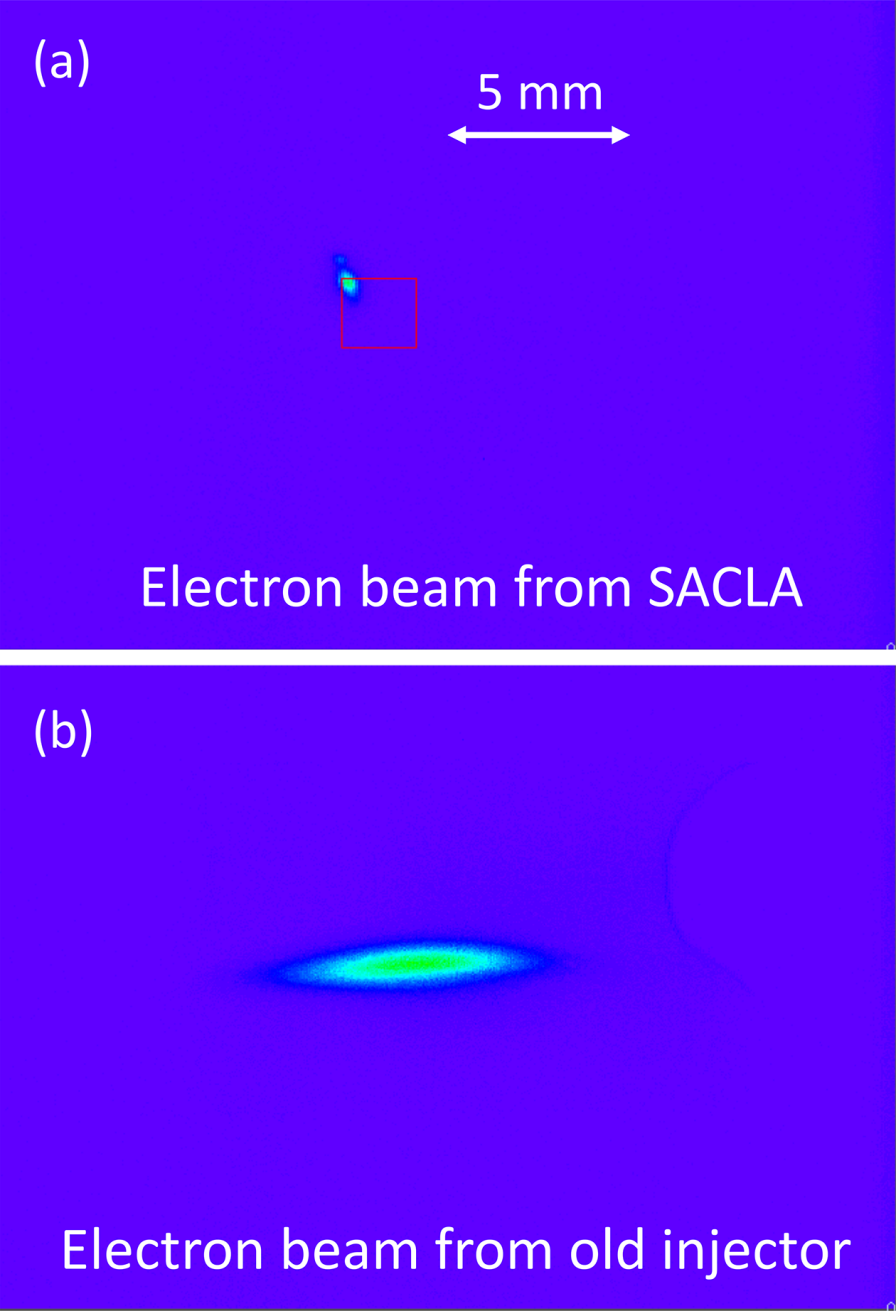
Profiles of the injected beams from SACLA (*a*) and old 8 GeV synchrotron (*b*).

Fig. 6 ▸ displays the profiles of the injected electron beam observed on a screen installed near the injection point of the SPring-8 storage ring. Compared with the electron beam from the old 8 GeV booster synchrotron, the beam from SACLA is significantly smaller, indicating a much lower beam emittance. While the emittance of the electron beam of SACLA is typically 0.1 nm rad, it deteriorates by one order of magnitude after XSBT, increasing to around 1 nm rad. This deterioration primarily comes from radiation excitation in the second half of XSBT. Nevertheless, even with this degradation, the emittance still sufficiently meets the 10 nm rad requirement for SPring-8-II, hence the second half of XSBT is used as is.

#### 4.1.5. Reliable electron gun system

In the beam injection from the SACLA linac to the SPring-8 storage ring, one of the most important issues for the reliable and steady beam operation is the reduction of the downtime in case of electron gun system failure, such as emission degradation of the CeB_6_ thermionic cathode, an accident of the high-voltage gun circuit, and a vacuum leakage of the gun chamber. Locating a spare electron gun next to the existing gun is not feasible because the passage of emission electron beams from the gun cathode through the non-straight line by kicking the beam results in the deterioration of the beam quality. Thus, placing such a spare gun adjacent to the existing gun cannot provide experimental users with a high-performance XFEL. To overcome this problem, the gun system was modified in 2022.

**Figure 7:**
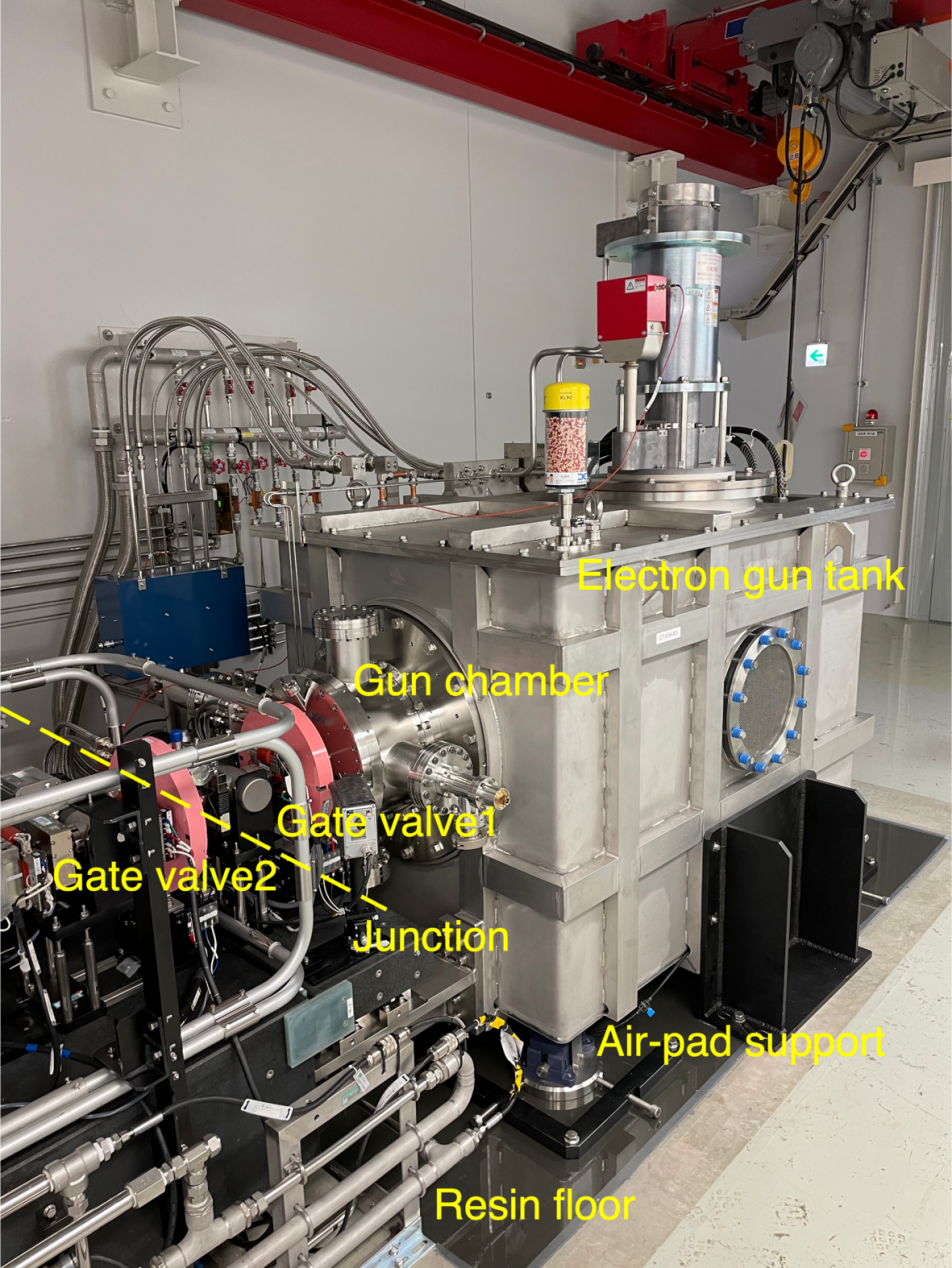
Photograph of the module-typed gun systems in SACLA. The junction of the electron gun tank and the injector beamline is indicated by a dashed yellow line.

The entire gun system, including the high-voltage tank and the CeB_6_ cathode, was constructed as an exchangeable module to facilitate quick recovery from any fatal problem that may occur in the gun system. Fig. 7 ▸ shows the module-typed gun systems installed in the SACLA accelerator tunnel. The system was designed so that the gun tank is exchanged with keeping the vacuum of the gun chamber and the injector beamline using two gate valves and a junction duct with a vacuum pumping port. The gun tank is connected to the beamline with a high position accuracy of 0.1 mm using four air-pad supports on a flat resin floor. The preparations of the spare gun, *i.e.* the cathode exchange, generation of the ultra-high vacuum, the cathode pre-heating, the high-voltage conditioning, and quality verification of the emission current, and the beam emittance, is performed in the gun test stand which was equipped with the same environments as SACLA (Togawa, 2024 ▸).

**Figure 8:**
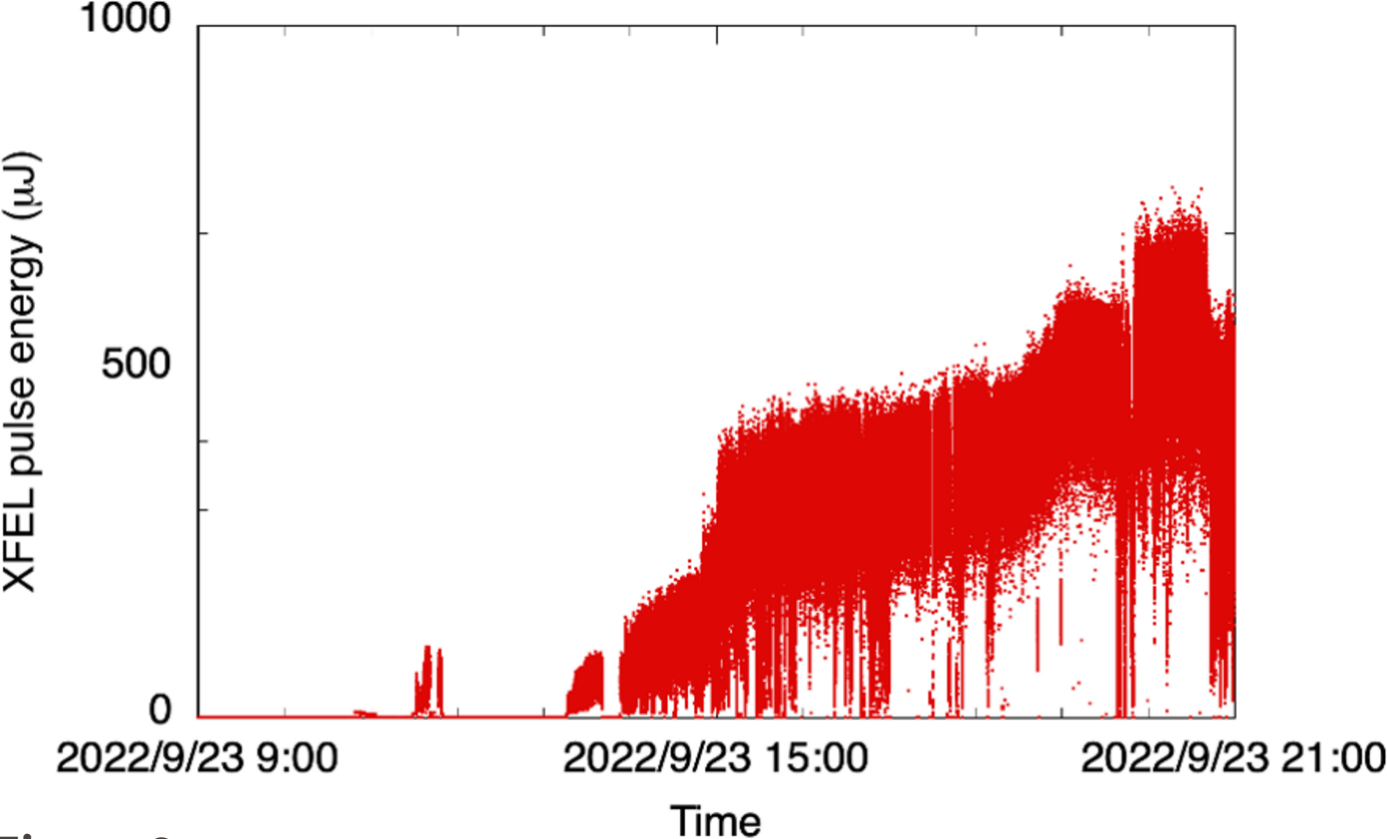
Example of the XFEL pulse energy recovery after the gun exchange. The starting point for the horizontal axis represents the time when the beam tuning was started after the gun exchange and the full scale is half a day.

The spare gun is always ready at the entrance of the accelerator tunnel in case required. Injection of the electron beam to the SPring-8 storage ring will restart within several hours after the start of the exchange work. SACLA needs additional time because finer beam tunings from the gun to the undulator line are required to recover the XFEL lights. Fig. 8 ▸ shows an example of the recovery of the XFEL pulse energy after the gun exchange. The XFEL pulse energy could be smoothly increased up to an intensity level of approximately 0.5 mJ in half a day with the help of machine-learning-based tuning (Iwai *et al.*, 2023 ▸).

### 4.2. Storage ring

#### 4.2.1. Optics design

**Figure 9:**
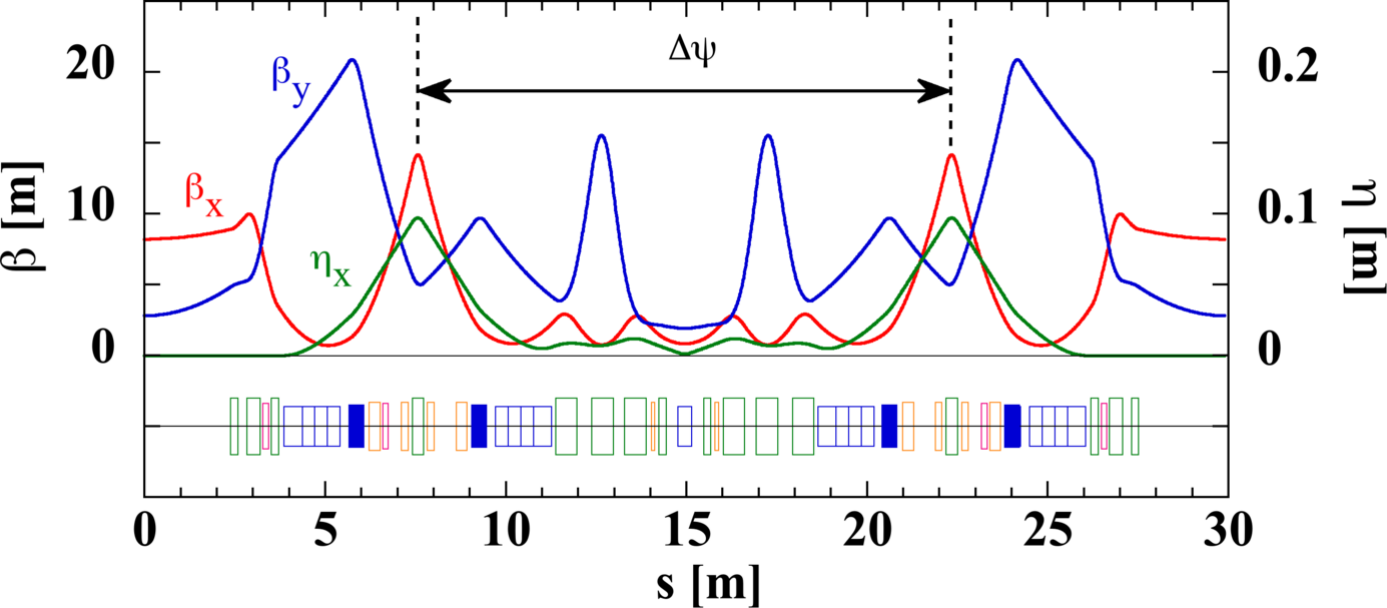
The normal cell of the SPring-8-II five-bend achromat lattice. The symbols β_*x*_ and β_*y*_ indicate the horizontal and vertical betatron functions, respectively, and η_*x*_ the dispersion function. The squares at the bottom represent dipole (blue), quadrupole (green), sextupole (orange) and octupole (red) magnets, and blue filled squares are DQ combined-function magnets.

To achieve the target performance specified in Section 2, we change the storage ring lattice structure from the present double-bend type to the multi-bend one with longitudinal gradient bending (LGB) magnets (Einfeld *et al.*, 1995 ▸; Nagaoka & Wrulich, 2007 ▸; Raimondi *et al.*, 2021 ▸). After various design works and simulation studies, we decided to employ a five-bend achromat lattice (Watanabe & Tanaka, 2023 ▸). Fig. 9 ▸ shows the optical functions and the magnet arrangement for one unit of the normal cell. The storage ring consists of 34 normal cells, 2 injection cells, 4 long straight sections (LSS) and 8 matching cells adjacent to each LSS (see below). Table 1 ▸ in Section 2 summarizes the main machine parameters of the newly designed SPring-8-II storage ring together with those of the present SPring-8 storage ring for comparison.

The linear and nonlinear lattice optimizations were carried out under the constraint of reusing the machine tunnel and keeping the source point of insertion device (ID) beamlines unchanged. The emittance was reduced as much as possible to keep the dynamic aperture (DA) and the momentum acceptance (MA) at acceptable levels for the off-axis beam injection and sufficient Touschek beam lifetime. Although the betatron phase difference between the two arc sections (see Fig. 9 ▸) is basically set to 3π and π for the horizontal and vertical directions, respectively, to cancel the dominant nonlinear effects due to the sextupoles, these phase differences are slightly detuned after carrying out the nonlinear optimization (Soutome *et al.*, 2016 ▸, 2017 ▸) and are finally set to 2.976π and 0.990π. The use of dipole–quadrupole (DQ) combined-function magnets increases the horizontal damping partition number from 1 to 1.39, which reduces the horizontal emittance. The ring has four LSSs and we are planning to install DWs in stages to further reduce the emittance to the level of 50 pm rad (Soutome *et al.*, 2022 ▸). We therefore took care to reduce the radiation loss by the dipoles to enhance the damping effects by DWs during user operation. Note that there have been a variety of promising approaches proposed towards fourth-generation light sources such as a combination of LGBs and reverse bends (RBs) that can provide significantly lower emittance than a multi-bend achromat lattice with one homogeneous bending magnet (Riemann & Streun, 2019 ▸). For SPring-8-II, we employ DWs for further reduction of emittance considering a use of DWs for user experiments as well as its capability of emittance compensation. One of features of fourth-generation light sources is that the emittance variation by changing undulator gaps during user operation can be significantly large compared with its small emittance (Hiraiwa *et al.*, 2022 ▸). Thus, a feed-forward adjustment of DW gaps is expected to be one of the options for stabilizing the light source performances such as brilliance and transverse coherence. We also plan to use DWs as high-energy X-ray sources above 100 keV with high penetration power, which are useful for the non-destruction observation of thick objects or the analysis of matters under high-pressure conditions.

**Figure 10:**
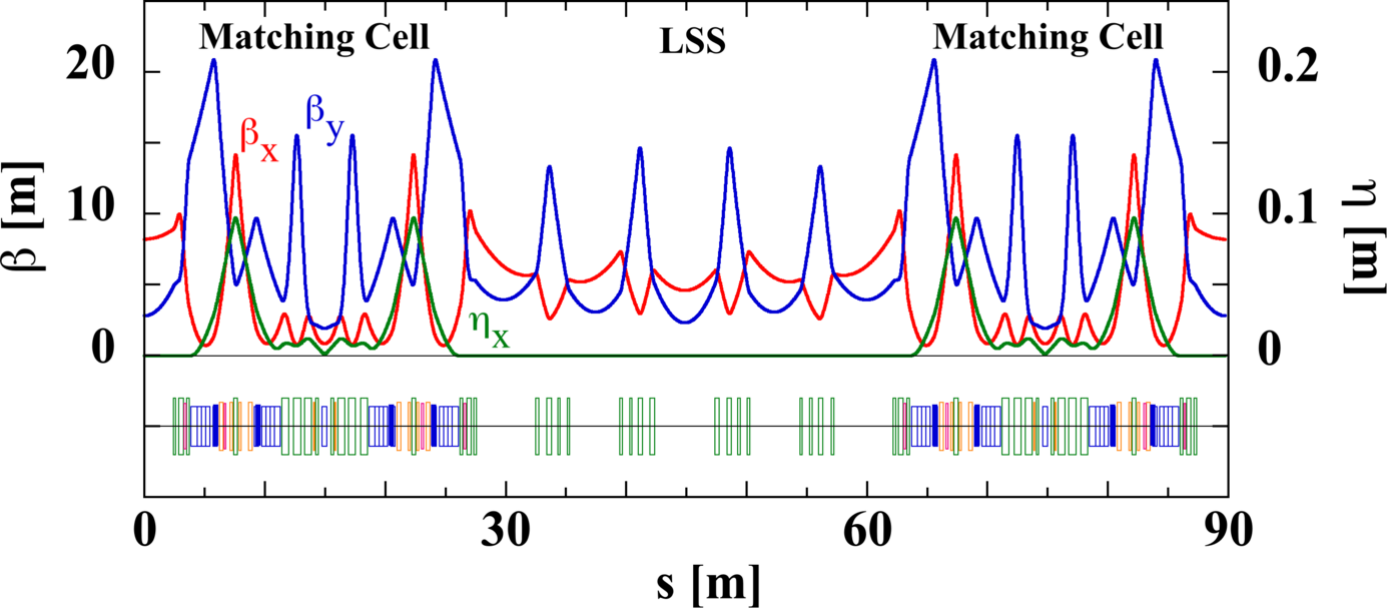
The optical functions and the magnet arrangement of the LSS and adjacent matching cells.

Fig. 10 ▸ shows the lattice design for the LSS and adjacent matching cells. The optics in the LSS was designed to avoid the generation of large natural chromaticities since there is no sextupole in this section. At the boundary with the normal cell, we imposed matching conditions to the betatron and dispersion functions up to the first order of the relative momentum deviation (Soutome *et al.*, 2022 ▸). This can be done by adjusting the quadrupole strengths in the matching cells. By adjusting both horizontal and vertical betatron phase advances to 2π over the LSS, the relative phase relation among the arcs in the matching cells where sextupoles are installed is unchanged. Then, even when the strength of the sextupoles in the arc changes arbitrarily, the off-momentum matching conditions at the boundary with the normal cell are automatically satisfied (Raimondi & Liuzzo, 2023 ▸). The off-momentum matching conditions are useful for elongating the Touschek beam lifetime through the MA enlargement. We note that as long as these matching conditions are satisfied, the optics of the LSS can be customized locally according to users’ requirements without degrading the beam performance.

**Figure 11:**
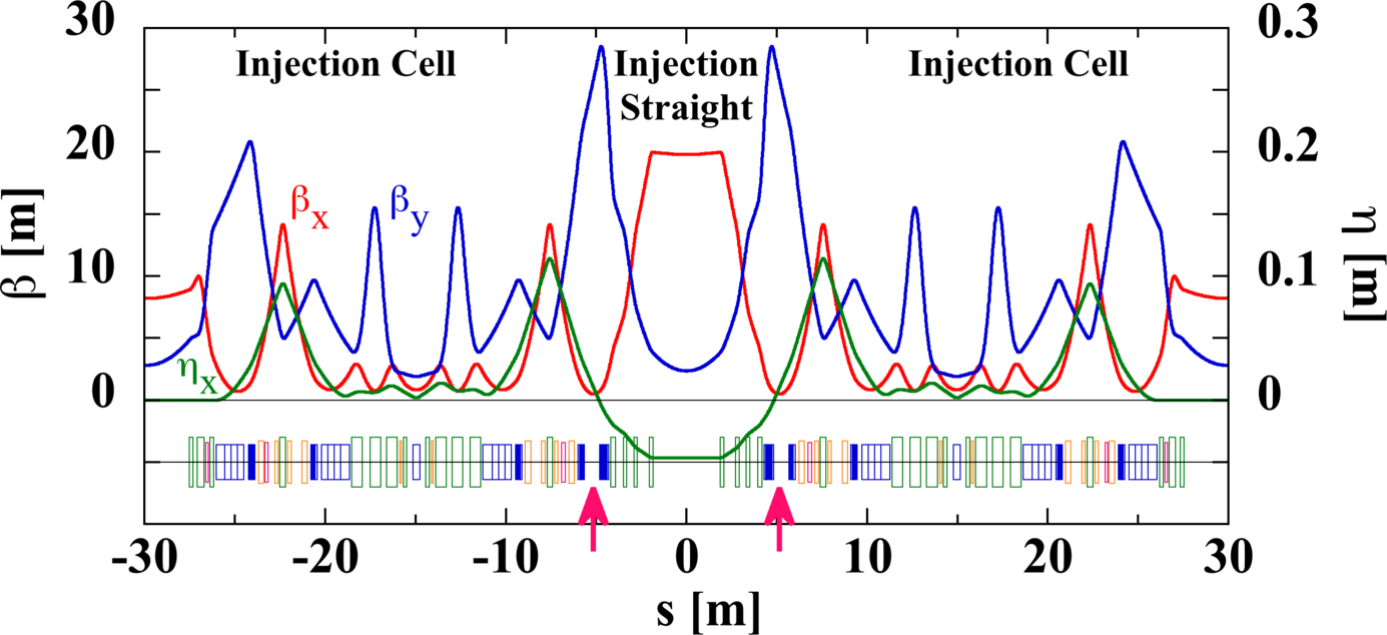
The optical functions and the magnet arrangement of the injection section. The arrows indicate the position of pulsed bump magnets.

A high-quality beam from the SACLA linac is injected into the storage ring with an advanced off-axis beam injection scheme using a pair of identical pulsed bump magnets. Fig. 11 ▸ shows the lattice for the injection section. To operate this scheme, two bump magnets are placed across the injection point so that the horizontal betatron phase difference is π, and the injection straight was designed to have a high horizontal betatron function of about 20 m. Since there are no sextupoles between the bump magnets, the bump orbit is not perturbed by nonlinear fields and is perfectly closed in principle. The quadrupoles between the bump magnets can be tuned independently for adjusting the betatron phase advance precisely to π.

**Figure 12:**
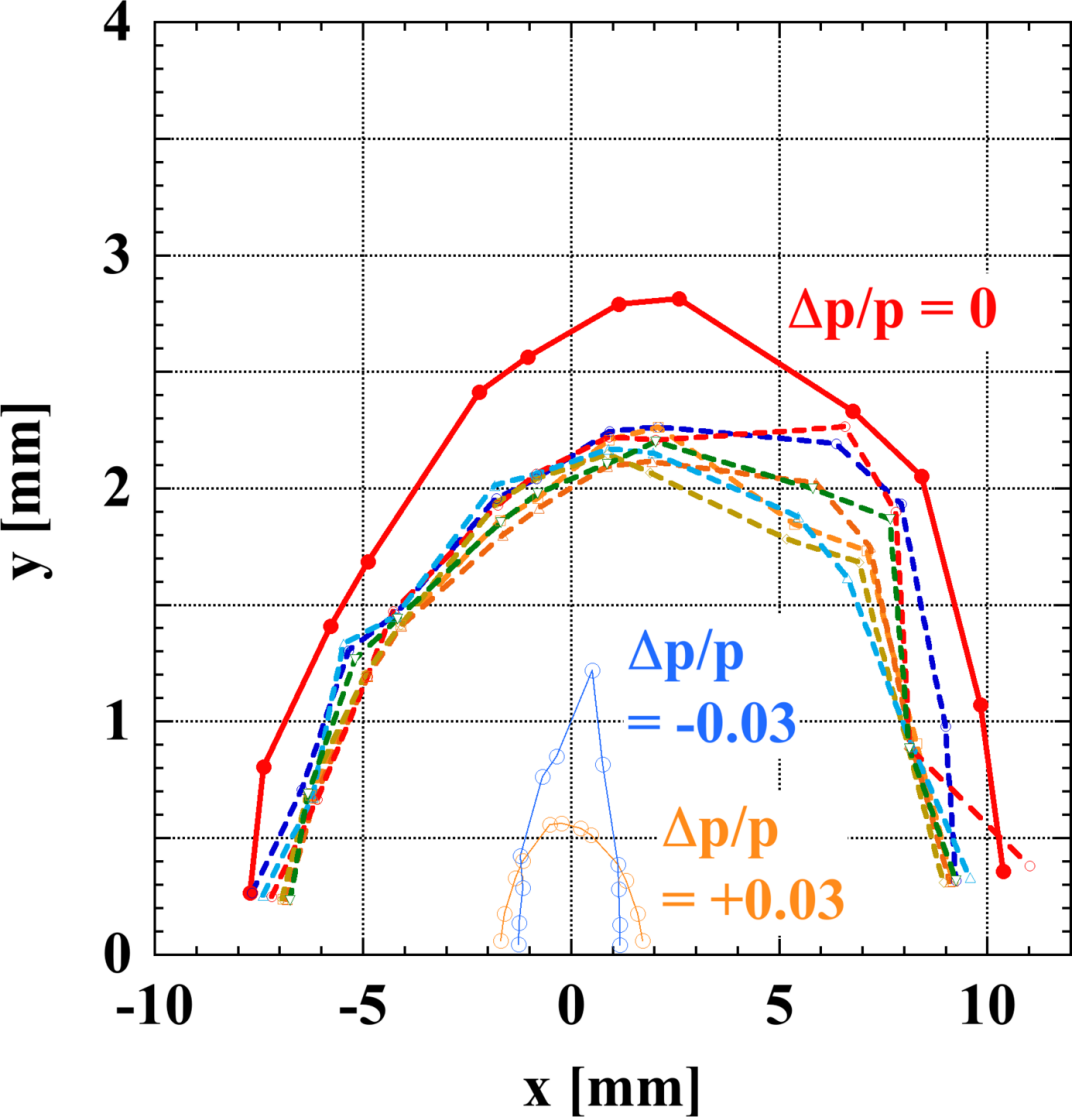
The dynamic apertures (DAs) for 1000 turns at the center of the injection straight where β_*x*_ = 19.8 m and β_*y*_ = 2.4 m. The solid red curve and dashed curves are on-momentum DAs for an ideal ring without errors and for the ring with sextupole misalignment of σ = 15 µm r.m.s. with the cutoff at ±2σ, respectively. Off-momentum DAs for ±3% are also shown (orange and light blue). The energy oscillation is taken into account by turning on the RF voltage at 5 MV.

The on- and off-momentum matching conditions are met at the boundary with the normal cell as in the matching cell for the LSS. The dipole field distribution within the injection cell is used as a tuning knob for the off-momentum dispersion matching and thus the dipole fields in this section are different from those in other normal cells. Fig. 12 ▸ shows the DA at the center of the injection straight. For the tracking simulations, we used an in-house code *CETRA* (Schimizu *et al.*, 2001 ▸) which can perform symplectic integration based on the Hamiltonian, and the results were cross-checked by using the code *ELEGANT* (Borland, 2000 ▸). Since the beam is injected at around *x* = −4 mm in this figure, we see that the DA is large enough for accepting the beam. The Touschek beam lifetime (Piwinski, 1998 ▸) was estimated by calculating the local momentum acceptance. At the stored current of 200 mA, the Touschek beam lifetime is expected to be about 10 h for a multi-bunch filling mode under the condition that a bunch current is approximately 0.1 mA and the horizontal and vertical emittance values are 111 pm rad and 10 pm rad, respectively, without considering the damping effect of insertion devices. For this bunch filling mode, the intrabeam scattering effects have also been calculated (Bane, 2002 ▸), and show an increase in the horizontal emittance of about 5% and in the energy spread of about 1%. This suggests that the emittance could be further increased with a high bunch current of 1 mA.

We note that by changing the strength of the quadrupoles, the dispersion at the normal straight is controllable. As discussed in detail by Hiraiwa *et al.* (2022 ▸), the emittance variation caused by the change of ID gaps during user operation can be well suppressed by optimizing the amount of dispersion leakage in the ID straight section. The suppression of the emittance variation, or the coherent portion fluctuation, will be important for users who need a stable photon beam for coherence-related experiments. In the present lattice design, it is possible to leak the dispersion in the ID straight section up to about 15 mm while maintaining the LSS as achromatic and keeping the natural emittance and the dynamic aperture at acceptable levels. We will start beam commissioning with the achromat optics, and, after sufficient progress made by machine tuning, we will consider switching to the non-achromat optics. If necessary, we will also adopt slow feedback of using the DW fields as an additional tuning knob for balancing the damping and excitation effects. The variation of the emittance is expected to be about 1% or less.

#### 4.2.2. Magnets

The main specifications for the lattice magnet are listed in Table 2 ▸. The five-bend lattice of SPring-8-II comprises four longitudinal gradient bending (LGB) and one normal bending (NB) magnets in addition to four dipole–quadrupole (DQ) combined-function, sixteen quadrupole (Q), ten sextupole (S), and four octupole (O) magnets in each normal cell.

The LGB and NB magnets are manufactured with permanent magnets (PMs) to reduce the power consumption. The PM-based magnets also allow to eliminate machine troubles caused by failures of power supply or cooling water, reduce vibrations originating from cooling water flows, and avoid hardware complexities due to coil and cable placements. We have developed PM-based LGB and NB magnets so that (i) magnetic fields on electron beams can be smoothly adjusted by about 10% using a movable iron plate for magnetic shunt, (ii) long-term drift of magnetic field such as radiation-induced demagnetization can be monitored in an open slot in a return yoke, and (iii) the temperature dependence of the permanent magnet material is reduced to less than ±5 × 10^−5^ K^−1^ by use of a magnetic shunt with Fe–Ni alloy (Watanabe *et al.*, 2017 ▸; Taniuchi *et al.*, 2020 ▸). By using Sm_2_Co_17_ as the permanent magnet material with the high radiation resistance, we carefully designed the LGB and NB magnets in such a way that the iron yokes are placed between the permanent magnet and the beam axis for suppressing the demagnetization due to the radiation and smoothing out the magnetic field distribution on the beam axis. Also, the permeance coefficients of the PM materials are adjusted higher than unity in most magnet regions (Watanabe *et al.*, 2017 ▸; Taniuchi *et al.*, 2020 ▸). Each LGB magnet consists of four segments to produce the step-function-like longitudinal magnetic field distribution, where iron poles near the beam axis come in a nose structure for a smooth field transition between the segments (Watanabe *et al.*, 2017 ▸).

DQ combined-function magnets are composed of four asymmetric poles to meet the requirements of dipole and quadrupole fields with a sufficiently good field region. In our design, we apply a common magnet design for two different magnet specifications. Although the ratio of bending and quadrupole components differs for each family of DQ combined-function magnets, we provide the required fields from the commonly designed magnet by changing the lateral magnet displacement against the beam axis and the excitation currents.

Quadrupole, sextupole and octupole magnets are electromagnets composed of laminated adhesive silicon steel sheets with a thickness of 500 µm. The laminated core is advantageous for suppressing manufacturing errors in a cost-effective manner, and for enabling quick magnetic field adjustments when required. The bore diameters of the quadrupole, sextupole and octupole magnets were determined by considering clearances between the vacuum chambers and the magnet poles. All the quadrupole, sextupole and octupole magnets are water-cooled with hollow conductors. The current densities are suppressed to less than 5 A mm^−2^ considering power consumption and machine failures. The temperature increase of the cooling water is estimated to be 10 K or less.

The main specifications for the correction magnets are listed in Table 3 ▸. Some of the sextupole and all of the octupole magnets are combined with a steering function that provides both vertical and horizontal kicks. The maximum deflecting angle ranges between 0.45 and 0.15 mrad for each steering magnet as listed in Table 3 ▸. All the octupole magnets have auxiliary coils to provide beam steering and skew quadrupole functions that can be switched by cable connection. The maximum kick angle of the steering function and the skew quadrupole field of the octupole magnet are 0.20 mrad and 0.29 T m^−1^, respectively. Two pairs of independent steering magnets will be installed on both sides of an undulator; one pair is to compensate for a small but fast beam deflection coming from undulator error fields via feed-forward correction, and the other is to correct long-term relatively large drift of the photon beam axis due to deformation of the accelerator buildings and other reasons. The small-angle correction will be conducted using air-core steering magnets, while the large-angle steering magnets will be made of a massive iron core.

**Figure 13:**
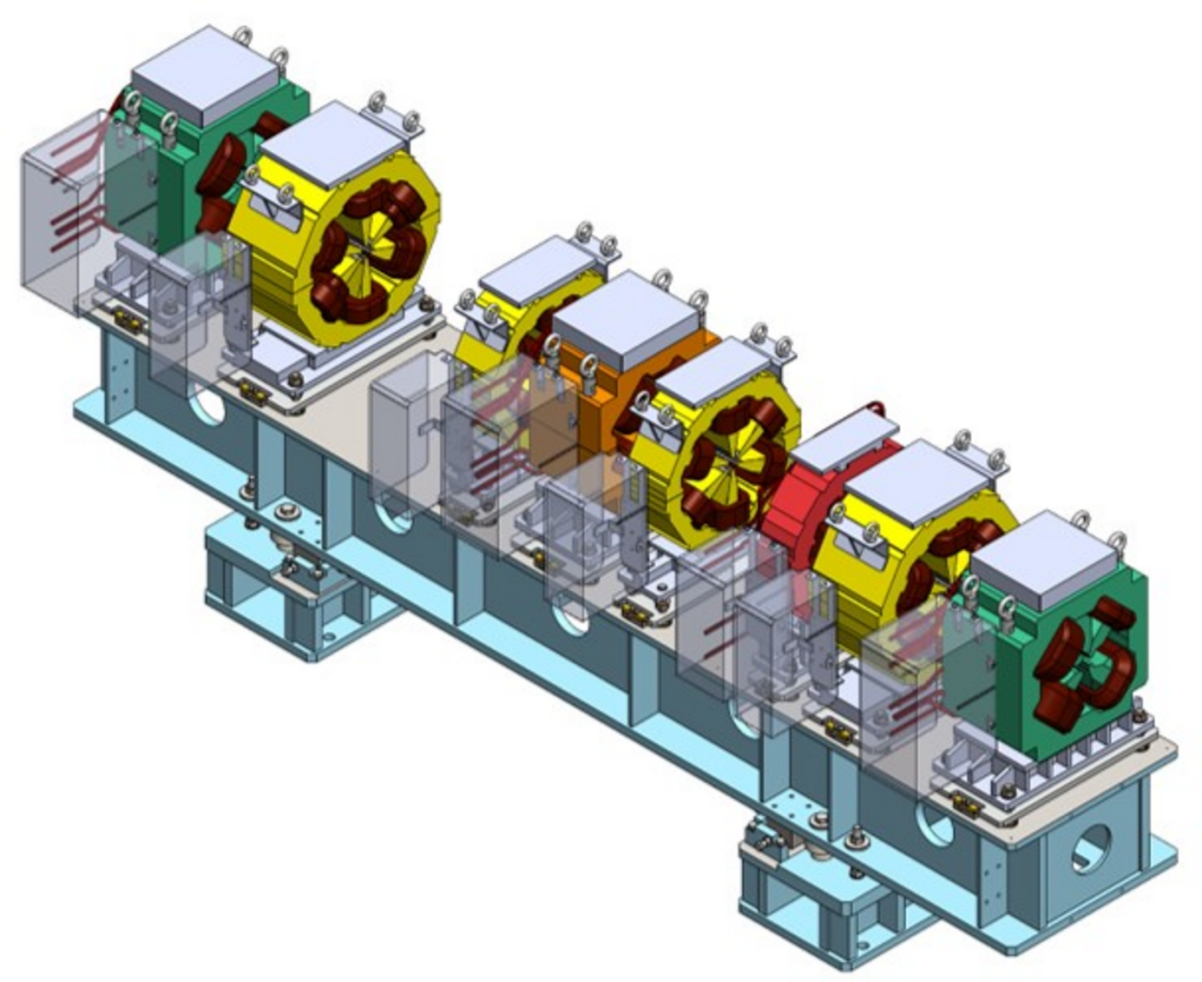
Three-dimensional drawing of the multipole magnets and common girder in the straight section.

There are 11 common girders in each cell and the magnets are precisely aligned on the girders. Fig. 13 ▸ shows a three-dimensional image of the common girder with multipole magnets mounted. The on-girder alignment will be carried out by the vibrating wire method (Fukami *et al.*, 2019 ▸), where a wire is excited by an alternating current (AC) to detect the off-axis wire position in a magnet. The method allows to align the magnetic centers in a straight line without any fiducialization. The girder-to-girder alignment will be carried out using laser trackers. The tolerance of the on-girder alignment and girder-to-girder alignment are described in Table 4 ▸.

Each family of quadrupole and sextupole magnets between cells is connected in series with a single electric power supply. For power supplies of the quadrupole and sextupole magnets, we have implemented a digital feedback control system using a field-programmable gate array (FPGA). This digital control system can be customized to satisfy various requirements. The power supply also features on-site adjustment of the feedback control parameters, making it suitable for various magnets. We also developed a high-precision current-detection circuit equipped with a 24-bit analog-to-digital converter to ensure a high current stability of less than 10 p.p.m. Some individual quadrupole magnets are connected to auxiliary power supplies for individual adjustment, which allows for compensating betatron function distortion and other purposes, such as beam-based alignment (BBA).

#### 4.2.3. Vacuum system

**Figure 14:**
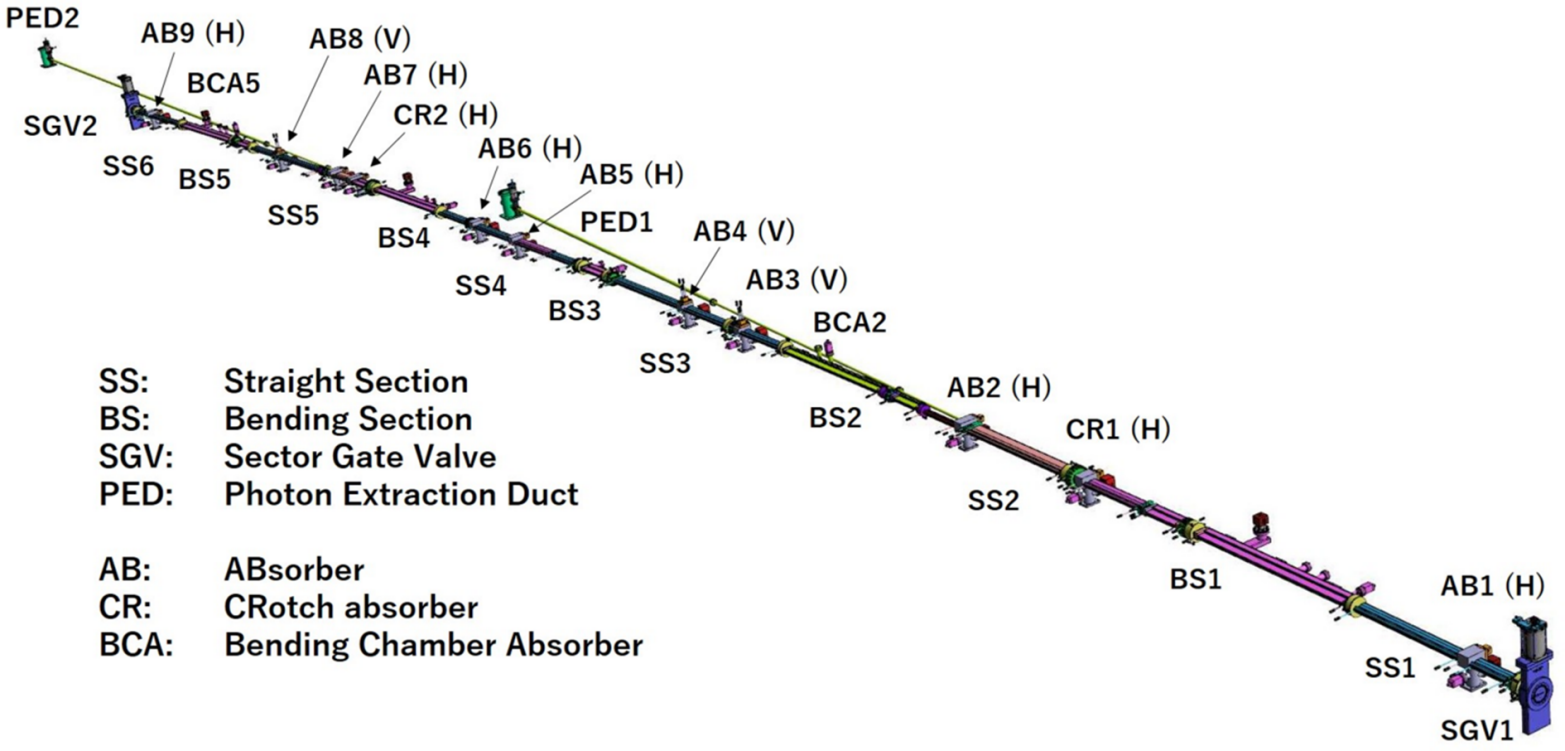
Vacuum system of the normal cell of SPring-8-II.

The vacuum system is designed to accommodate the narrow bore magnets, while ensuring ultra-high vacuum along the ring for long enough lifetime and low coupling impedances for stable beam operation. Fig. 14 ▸ shows the vacuum system for the normal cell with a total length of 26 m. It consists of 11 chamber pieces, 13 discrete photon absorbers equipped with vacuum pumps, and sector gate valves at both ends.

**Figure 15:**
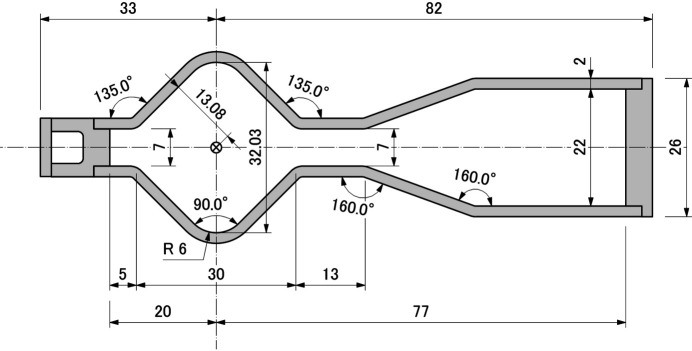
Cross-sectional shape of the new straight section chamber.

The vacuum chamber is made with 2 mm-thick stainless steel to assure clearance against the narrow bore magnets. The cross-sectional shape of the straight section chamber is designed to be a rhombic shape as shown in Fig. 15 ▸, which helps reduce the coupling impedance on the inner surface of chambers without copper plating. The design also contributes to suppress both manufacturing cost and time, which support the key concepts of SPring-8-II.

**Figure 16:**
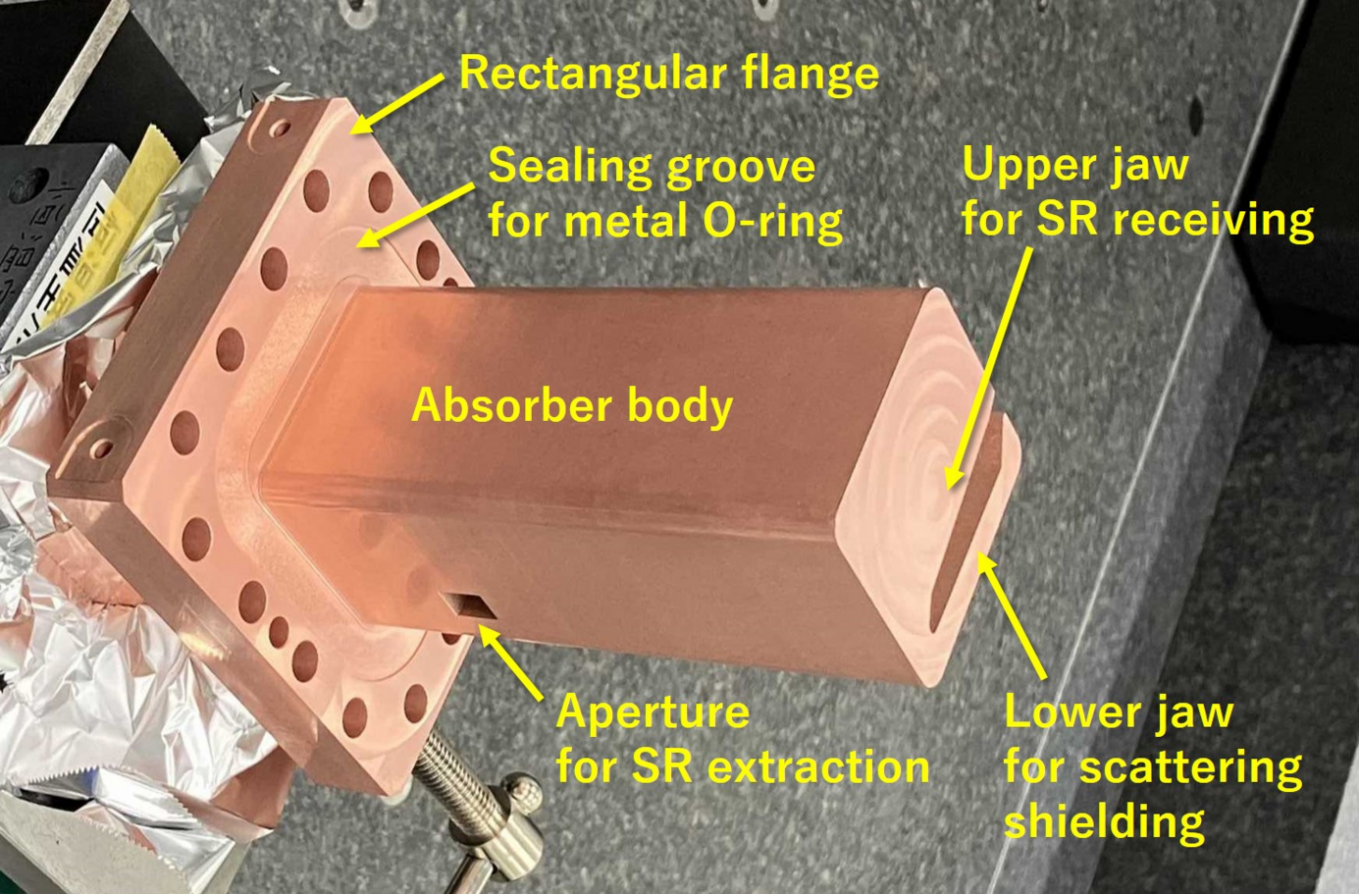
Prototype of a horizontal insertion type photon absorber made of CuCrZr.

The 13 discrete photon absorbers are composed of two crotch absorbers (CRABs) with an aperture for extracting synchrotron radiation, nine normal absorbers (ABs), and two bending chamber absorbers (BCAs) installed at the downstream end of two of the five bending sections. The photon absorber is designed to confine most of the scattered radiation from the irradiated SR inside the shielding structure. This structure is designed to confine the source of photon stimulated desorption (PSD) gas to the inside of the shielding structure and to reduce the vacuum conditioning time in the beam commissioning period of the storage ring. The material of the photon absorber is changed from Glidcop to a more common material CuCrZr. A flange-integrated structure that eliminates the risk of vacuum leakage is also adopted. For most of the photon absorbers that do not interfere with the photon extraction ducts, horizontal insertion type devices are used, because they have excellent cooling efficiency and a simple cooling piping structure. Fig. 16 ▸ shows a prototype of a horizontal insertion type photon absorber.

Since the narrow cross-sectional vacuum chambers reduce the pumping conductance, a small cartridge-type NEG pump and a sputtering ion pump (SIP) are installed near each photon absorber to efficiently evacuate the PSD gas generated during beam operation (Oishi *et al.*, 2016 ▸; Tamura *et al.*, 2019 ▸). Cold cathode gauges (CCGs) are installed near the photon absorbers downstream of each bending section.

To accommodate the tight installation schedule, the vacuum chamber is pre-baked outside the tunnel and only the NEG is reactivated without baking once installed in the tunnel. The pre-baking process can be performed without magnets, which facilitates the installation of baking heaters and heat insulators, and improves the quality of baking operations such as temperature control. Another advantage of this scheme is that SIP activation, NEG activation and CCG activation can be performed during pre-bake to confirm normal operation of the equipment prior to installation in the tunnel.

CCG with an internal electrode cleaning function is used as a vacuum gauge to eliminate the risk of filament breakage and to improve maintainability. Also, a new NEG reactivation scheme without any pump such as a turbo molecular pump or a SIP is under investigation to simplify the conventional NEG reactivation scheme using an external pump.

All the vacuum designs have been verified from viewpoints of collective effects and thermal stress analyses. The growth rate of the transverse coupled bunch instability (TCBI) for the stored current of 200 mA is estimated to be around 5 ms^−1^, which is small enough against the SPring-8 bunch-by-bunch feedback (BBF) system that provides the damping rate of 10 ms^−1^. The estimation of the TCBI growth rate is conducted under a conservative assumption of zero chromaticities, thus the designed vacuum components are proven to be appropriate for possible operating conditions of SPring-8-II. Heat loads on the vacuum chambers are estimated to be under 20 W m^−1^ for a filling pattern of evenly filled 406 bunches of 0.5 mA as an example. By optimizing the water cooling channels and chamber support structures, we confirmed that the resulting thermal stresses on vacuum chamber materials are well controlled below the allowable stress.

#### 4.2.4. Beam diagnostic system

The goal of the beam diagnostic system for SPring-8-II is to secure the performance of the electron beam as designed and to provide high-quality photon beams to users with ultimate stability. To address the requirement for the photon beam as well as the electron beam, we build a precise and stable electron beam position monitor (BPM) system, a bunch-by-bunch feedback (BBF) system to suppress beam instabilities and measure the betatron tune as well, a DC current transformer (DCCT) for a stored current monitor, a bunch-by-bunch current and phase monitor for the top-up operation, an X-ray pinhole camera (XPC) or an X-ray Fresnel diffractometry (XFD) for beam emittance diagnostics, and a novel X-ray BPM (XBPM) that can resolve the central core of the undulator photon beam. In this section, we briefly introduce each beam diagnostic component.

**Figure 17:**
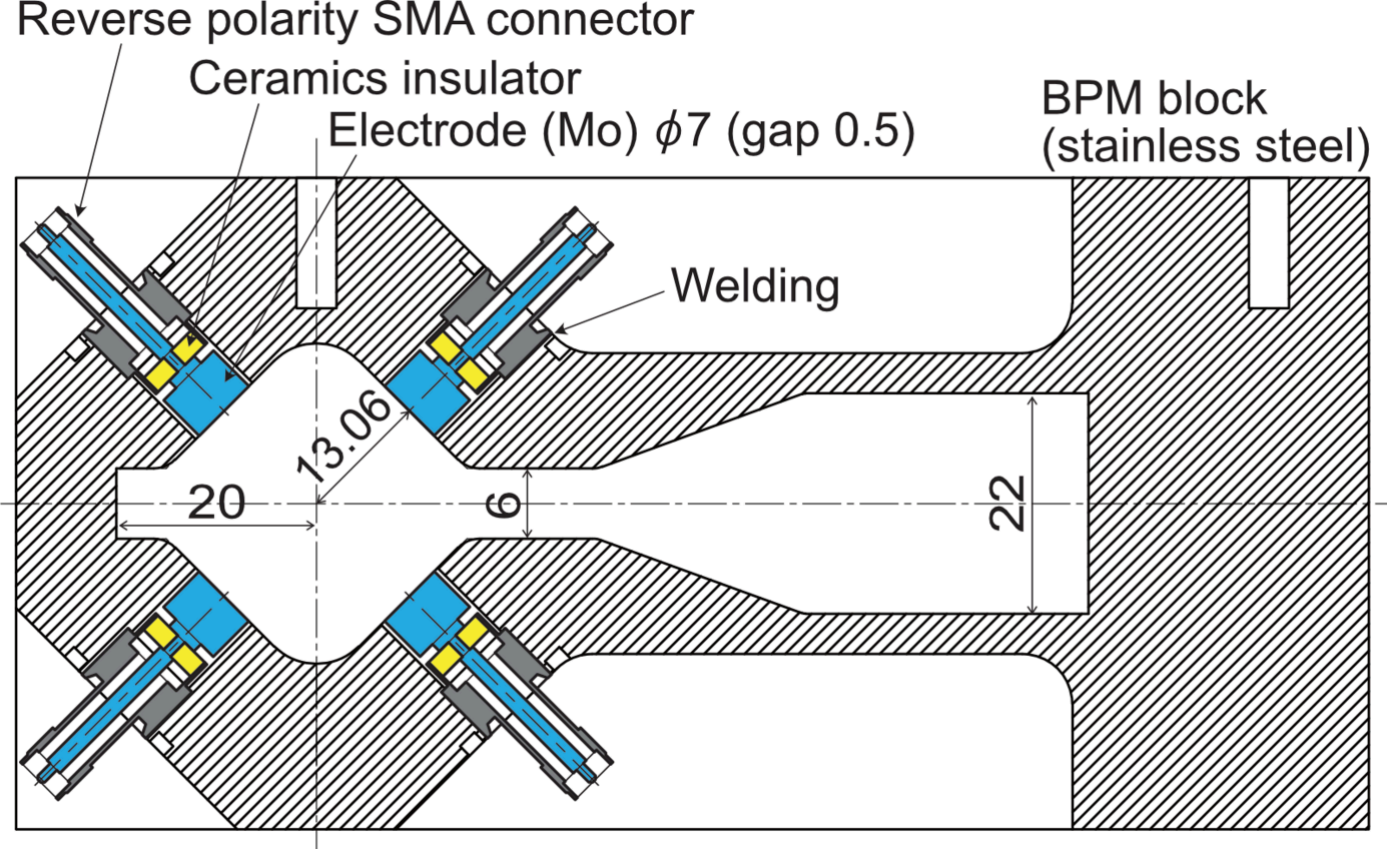
Schematic drawing of the BPM head and button electrodes. The unit of dimensions is mm.

The electron BPM features a stable closed orbit distortion (COD) measurement capability for user service and a precise single-pass (SP) measurement resolution for beam commissioning. The requirement for the stability of the COD-BPM is within 5 µm peak-to-peak for one month. The SP-BPM resolution is demanded to be 100 µm s.t.d. for 0.1 nC single-bunch. We have developed a button-type BPM system that can satisfy these requirements. A schematic drawing of the BPM head and button electrode is shown in Fig. 17 ▸. The design of the button electrode is described by Masaki *et al.* (2016 ▸). Although the shape of the vacuum chamber was changed, the sensitivity of each electrode to the beam was estimated to be comparable with the previous design described by Masaki *et al.* (2016 ▸). The BPM signal is transmitted by radiation-resistant cables (Fujita *et al.*, 2015 ▸; Maesaka *et al.*, 2018 ▸) and processed by versatile MicroTCA.4-based electronics (Maesaka *et al.*, 2019*b* ▸) which can calculate both COD-BPM and SP-BPM data in parallel. The BPM offsets will be calibrated against the magnetic center of a nearby quadrupole magnet by applying the conventional beam-based alignment method that relies on the beam orbit response. Based on the betatron tune shift, research is also underway on the offset calibration of BPMs to nearby sextupole magnets (Takano *et al.*, 2022 ▸).

A prototype BPM system was installed in the present SPring-8 storage ring for performance verification. The resolution of the SP-BPM was confirmed to be well below 100 µm s.t.d. for a 0.1 nC single-bunch beam and the stability of the COD-BPM was evaluated to be 5 µm peak-to-peak for one month (Maesaka *et al.*, 2019*b* ▸). Thus, the performance of the BPM system is sufficient for SPring-8-II. The BPM system developed for SPring-8-II, including the button electrodes, the radiation-resistant cables and the MicroTCA.4-based electronics, has also been implemented in NanoTerasu (Maesaka *et al.*, 2020 ▸) as the workhorse of the beam diagnostic instrument of the storage ring, contributing to the accelerator commissioning and the beam optimization towards user operation (Ueshima *et al.*, 2024 ▸).

The BBF system cures collective instabilities and measures the betatron tune. The growth rate of transverse instabilities is estimated to be approximately ten times greater than the present SPring-8 storage ring. The present transverse BBF system (Nakamura & Kobayashi, 2005 ▸) has sufficient gain to suppress the beam instabilities anticipated in SPring-8-II and also has a tune measurement function with a sufficient resolution of 10^−4^. The SPring-8-II storage ring would also suffer from a longitudinal coupled-bunch instability coming from higher-order modes in the acceleration cavities. A kicker for the longitudinal BBF has already developed (Nakamura, 2011 ▸; Masaki *et al.*, 2013 ▸) and the combination of the present BBF electronics and the longitudinal kicker is estimated to be sufficient to mitigate the longitudinal instability anticipated in SPring-8-II. Thus, the BBF system is ready for the SPring-8 upgrade. However, since the present BBF signal processor is becoming difficult to maintain, we are developing new electronics with RFSoC from AMD (former Xilinx) (Zynq UltraScale+ RFSoC, https://www.xilinx.com/products/silicon-devices/soc/rfsoc.html) to replace the old processor. All the current kickers and pickups for the transverse and longitudinal BBF will be also redesigned and replaced to be compatible with the new electronics and to match the vacuum chamber aperture of SPring-8-II.

The DCCT stored current monitor is demanded to have a measurement range of more than 200 mA and a high current resolution of 1 µA for stable top-up injection. The DCCT head and its front-end electronics of Bergoz instrumentation (https://www.bergoz.com/) satisfy the requirements. We have already developed a similar DCCT system for NanoTerasu (Maesaka *et al.*, 2020 ▸), which includes a vacuum chamber housing a DCCT head, and confirmed the sufficient performance with an actual electron beam. Therefore, a similar DCCT system is expected to satisfy the demand for SPring-8-II.

A bunch-by-bunch current and phase monitor system is also important for the top-up operation to maintain properly the bunch fill pattern and to ensure that the injection timing is within the longitudinal injection aperture of the storage ring. We employed a similar pickup electrode as the BBF system and a high-speed digitizer based on MicroTCA.4 having a sampling rate of 10 GS s^−1^ developed by Teledyne SP Devices (https://www.spdevices.com/). This system has been confirmed to have a bunch current resolution of approximately 2 µA s.t.d., corresponding to 10 pC, and a bunch phase resolution of less than 1° s.t.d. for a 0.1 mA bunch, corresponding to a time resolution of 5 ps. Thus, the bunch-by-bunch current and phase monitor has demanded performance for SPring-8-II.

An X-ray pinhole camera (XPC) will be employed for precise emittance diagnostics and installed into one of the dipole radiation beamlines to measure the source beam size (Takano *et al.*, 2015 ▸). When the natural emittance is reduced to 50 pm rad with an *XY* emittance coupling ratio of 10%, the horizontal and vertical beam sizes at the first dipole magnet for each cell are calculated to be 12 µm and 9 µm (s.t.d.), respectively. We optimized the configuration of the XPC with the radiation from this dipole magnet and the achievable beam size resolution was expected to be 5 µm. Consequently, the beam size can be measured by the XPC with a sufficient resolution.

We have another option for ultra-low emittance diagnostics – X-ray Fresnel diffractometry (XFD) (Masaki *et al.*, 2015 ▸) developed at SPring-8, which has a much higher resolution than the XPC. The Fresnel diffraction pattern of monochromatic X-rays by a narrow single slit of several tens of micrometres is taken by an X-ray image sensor, and the beam size is evaluated from the contrast of the two-lobe image. The XFD measurement is applicable to both bending magnet and undulator radiation sources. The XFD can resolve a beam size down to 1 µm s.t.d. and hence it can be a good proof of the achieved emittance.

A reliable X-ray beam position monitor (XBPM) is the key to suppressing the optical axis fluctuations and stabilizing the photon beam. We have been developing a novel XBPM that can resolve the central core of an undulator photon beam (Kudo *et al.*, 2022 ▸). By using the new XBPM, the optical beam axis of undulator radiation can be accurately measured without contamination by radiation from adjacent dipole magnets. This kind of XBPM will be installed into X-ray beamlines that require stringent optical axis stability. We are planning to use the real-time XBPM data as an input of the beam orbit feedback system in addition to the electron BPM data to achieve the ultimate stability of the optical axis.

#### 4.2.5. RF system

**Figure 18:**
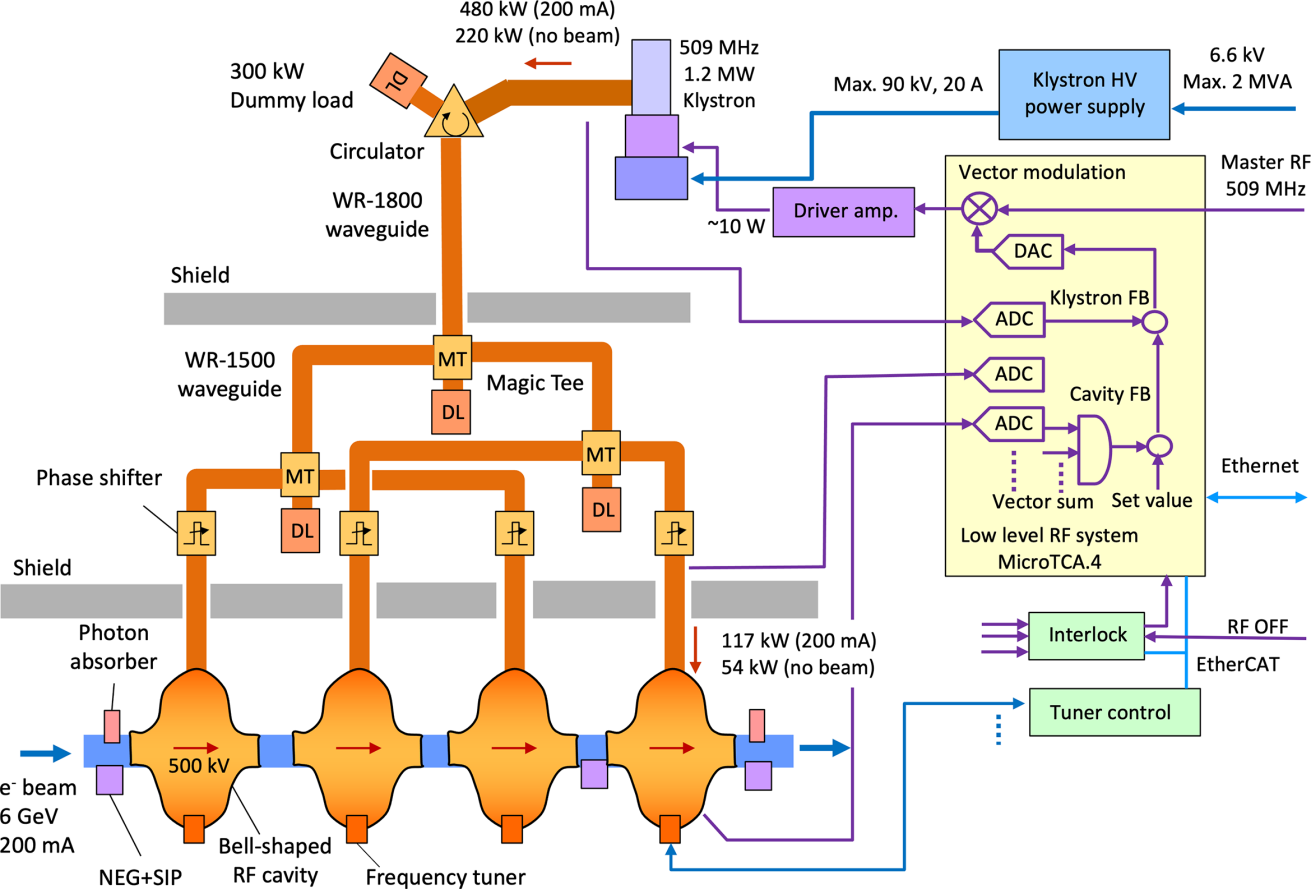
Diagram of the RF system in one RF section for SPring-8-II.

The role of an RF acceleration system of a storage ring is to generate a sufficient beam-accelerating voltage and compensate for beam-energy loss caused by synchrotron radiation. For SPring-8-II, we plan to reuse existing klystrons and related RF components of SPring-8 for suppressing the total accelerator construction cost and simplifying the construction procedures. Fig. 18 ▸ shows a diagram of the RF system. In the SPring-8-II storage ring, the required acceleration voltage is up to 8 MV, assuming an energy loss of 6 MeV per turn due to synchrotron radiation at bending magnets, undulators and damping wigglers. In the current SPring-8, there are four RF sections and each section has eight bell-shaped normal-conducting cavities, which generate the acceleration voltage of 500 kV each (Kawashima *et al.*, 2008 ▸; Ego *et al.*, 1997 ▸, 1998 ▸). In SPring-8-II, the number of cavities will be reduced by half to four cavities in each of the four RF sections, because the required voltage is half. The cavities can be tuned from the current RF frequency of 508.580 MHz to the new RF frequency of 508.764 MHz by adjusting the tuning plunger positions. These bell-shaped RF cavities with low shunt impedance are used to avoid coupled bunch instability due to a higher order mode (HOM) resonance of the cavities. The inner shape of each cavity and the plunger length are slightly changed one by one to shift the HOM frequencies so that they do not overlap (Ego *et al.*, 1997 ▸). Furthermore, if HOM is induced in one cavity, the HOM frequency can be shifted by changing the insertion length of two orthogonal plungers. A 1.2 MW klystron is used as the RF source of the cavities of each RF section. The required RF power is up to 480 kW for the nominal operating conditions. The anode voltage of the klystron is controlled according to the output power and the electrical power efficiency is more than 50%. If one of the four RF sections has some problems, it is possible to increase the cavity voltage of the other sections and continue operation with the three sections, since the klystrons still have a margin of output power. Because the size of the beam duct is reduced, the tapered beam ducts and photon absorbers are newly designed. They are located in front of and behind the cavities with NEG-composite vacuum pumps.

The low-level RF (LLRF) system that controls the amplitude and phase of the acceleration voltage uses a digital control system based on the MicroTCA.4 framework, which was constructed in 2017 (Ohshima *et al.*, 2017 ▸). With this system we control the phase and amplitude of the RF supplied to the driver amplifier for the klystron by applying in-phase and quadrature modulation to the reference RF. We measure the amplitudes and phases of the signals from the klystron output, the cavity input, cavity reflection and the pickup of the cavity using the under-sampling method. In order to stabilize the acceleration voltage of the cavities, two types of on-board feedback (FB) controls are built into this LLRF system. The klystron FB has a control bandwidth of a few kHz and suppresses the fast fluctuations caused by the high voltage ripple of the klystron power supply. The cavity FB suppresses slow variations in the order of a few Hz, such as changes in beam load and cooling water temperature. The cavity’s tuning plunger controller connects to LLRF via EtherCAT communications filed bus. It maintains the cavity resonance by adjusting the plunger position to keep the phase of the cavity’s input and stored RF. The interlock system has a function to cut off the RF output power when some of the equipment is troubled. When a fast stop signal, such as beam abort request signal, excessive reflected power from the cavity, *etc*., arrives, the RF switch is turned off within several micro-seconds. The source of the interlock is recorded, including the order of first arrival. Information on all such devices can be communicated to and controlled by the higher-level control system via an EtherCAT and Ethernet connection.

#### 4.2.6. Beam injection section

Challenges in beam injection for SPring-8-II are (i) off-axis injection into the narrower dynamic aperture, (ii) ‘transparent’ top-up operation without interfering with SR experiments, even with the much smaller stored beam than the present one, and (iii) meeting the requirements of a ‘green’ facility. Separation between the injected beam and the bumped stored beam orbit in the off-axis injection is limited within 5 mm. Transient stored-beam oscillation during injection should be one-third or less of the reduced beam size for assuring the transparency to SR experimental users. The DC septum magnets used for the present beam injection section need to be replaced to reduce the power consumption.

**Figure 19:**
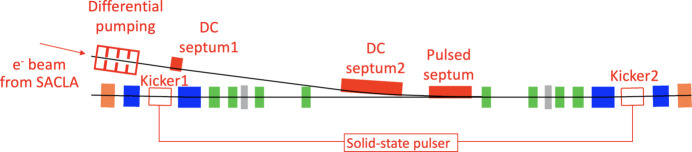
Schematic layout of the beam injection section for SPring-8-II.

**Figure 20:**
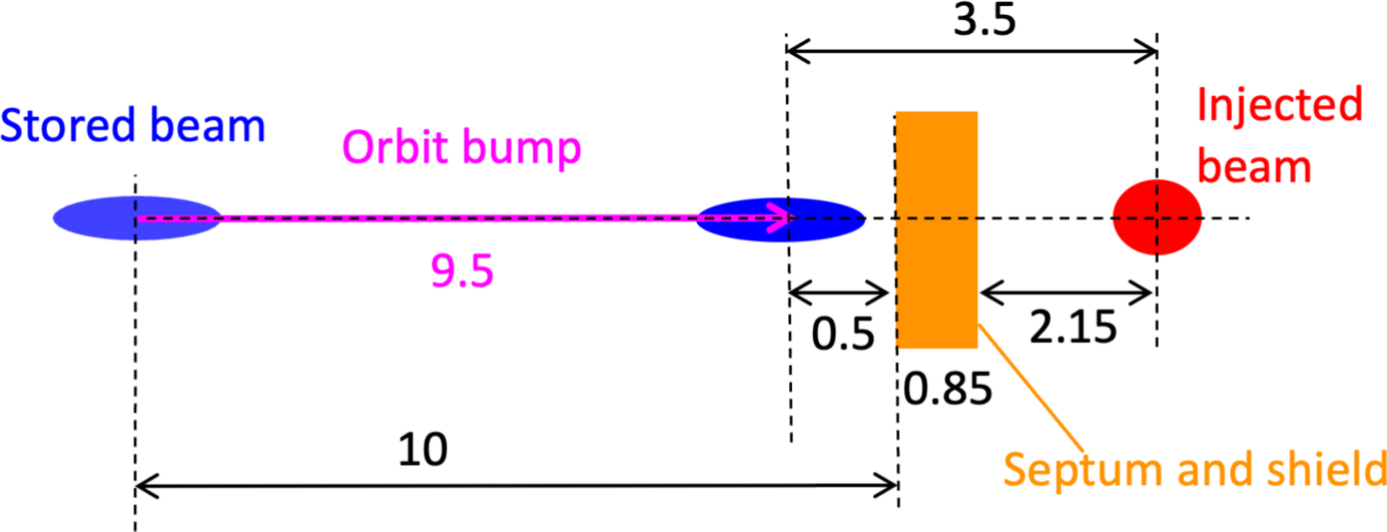
The transverse position of the injected beam and the stored beam at the beam injection point. Numbers without units in the figure represent millimetres. Parameters are subject to change with future design revisions.

The layout of the new beam injection section for SPring-8-II is schematically illustrated in Fig. 19 ▸. The low-emittance beam delivered from the SACLA linear accelerator passes through a differential pumping system, which links the beam transport line with a moderate vacuum condition to the ultra-high-vacuum section directly connected to the storage ring. Two DC septum magnets and one pulsed septum magnet deflect the injection beam to the beam injection point. Two identical kicker magnets driven by a common pulsed power supply are placed on the beam injection section of the storage ring to provide a closed orbit bump for beam injection. The transverse position of the injected beam and the stored beam at the beam injection point is illustrated in Fig. 20 ▸. The pulsed septum magnet is an in-vacuum thin eddy-current type, where the total thickness of the septum wall and the magnetic field shield is as small as 1 mm. The distance between the stored beam orbit and the septum wall is currently set to be 10 mm and the height of the pulsed orbit bump to be 9.5 mm. The distance between the injected beam and the bumped stored beam is currently set to be 3.5 mm to secure efficient beam injection into the dynamic aperture. The parameters of the magnets relevant to beam injection are summarized in Table 5 ▸.

For the DC septum magnets, we have designed and developed a permanent magnet (PM) based one where we solved the issues such as the suppression of fringe fields on stored beam, temperature dependence of the PM material, demagnetization, and fine tuning of the magnetic field on beam (Taniuchi *et al.*, 2020 ▸). The newly developed DC septum magnets will also contribute to the reduction of power consumption as the DC septum magnet is one of the most power consuming magnets in the existing storage ring.

An in-vacuum eddy-current septum magnet has also been newly designed and developed (Takano *et al.*, 2019 ▸). A 0.5 mm-thick septum and 0.35 mm-thick magnetic shields at the exit of the septum magnet enable the injection amplitude of about 3.5 mm while achieving the designated gap field of 1.4 T on the injection beam, enough low stray field on stored beam in the level of 10^−5^ T m or even less, and ultra-high vacuum (UHV) of the order of 10^−8^ Pa.

The key to successful transparent beam injection in our linear π-bump scheme is the closure of the injection orbit bump which is assured by equalizing the gap fields of the two kickers during the entire excitation period. Considering the present parameters of SPring-8-II relevant to the beam injection, a deviation of the gap fields between the two kickers should be controlled below 0.1% to suppress the transient oscillation of the stored beam to one-third or less of the stored beam size. The two kickers are driven in parallel by a common pulsed power supply so that the timing jitter of the pulser does not yield the timing difference in the kickers. The kicker magnet that enables the field deviation below 0.1% by introducing a laminated core of thin electrical steel sheets has been proposed and developed (Fukami *et al.*, 2022 ▸). The inner metallic coating on ceramic vacuum chambers for kickers is another key factor for the injection transparency because the eddy current in the conventional metallic coating can degrade the magnetic field inside the kicker chambers. For that, we have developed a ceramic vacuum chamber with longitudinal stripe Ti coating for the suppression of the eddy current. A prototype of a solid-state pulsed power supply for the kicker has been developed featuring a series of solid-state HV switches (IGBTs), a high-precision HV charger, and internal variable inductors for current balance correction in the parallel drive of two kicker magnets (Inagaki *et al.*, 2018 ▸).

#### 4.2.7. Insertion devices

Because of the shorter straight sections in SPring-8-II than those in the current SPring-8 by nearly 1 m, all the existing insertion devices (IDs) should be replaced with shorter ones. Most of them are the so-called in-vacuum undulators (IVUs), in which magnetic arrays are placed inside the vacuum chamber and the achievable magnetic field can be significantly higher than that of out-vacuum undulators. Although the narrower minimum gap available with the in-vacuum structure brings a great advantage, in particular for short-period undulators, IVUs based on a conventional design have several technical difficulties in terms of the manufacturing cost and limitation on the achievable performance. In particular, the former may be a critical issue in SPring-8-II, because we need to build more than 30 IVUs to fit into the shorter straight section. To proceed the SPring-8-II project within a limited budget, it is thus essential to establish a procedure to construct many IVUs in a cost-effective manner without sacrificing the performance. For this purpose, we established a new concept of IVU, referred to as ‘IVU-II’ (IVU for SPring-8-II) (Imamura *et al.*, 2024 ▸), which is based on three key technologies: force cancelation by monolithic multipole magnets, modularization of the magnetic array, and 45° inclined Halbach configuration. As of March 2024, seven IVU-IIs have been built, and one of them has been installed. We are planning to construct up to six IVU-IIs per year before starting the shutdown, so that all of the existing IVUs currently in operation in SPring-8 can be replaced during the shutdown period.

Besides the above-mentioned IVU-II as a standard ID for X-ray beamlines, we developed a new ID for soft X-ray (SX) beamlines, ‘helical-8 undulator’ (Tanaka, Seike *et al.*, 2023 ▸). It is composed of six magnetic arrays, and four outer arrays form a special magnetic circuit capable of switching the magnetic period (fundamental/double) mechanically. As a result, the helical-8 undulator can be selectively operated as a helical/figure-8 undulator to switch the polarization mode and thus is capable of switching the polarization state of SR among circular (left- and right-hand), vertical and horizontal ones. What should be emphasized is that it has an advantage that the on-axis heat load in the linear-polarization mode is much lower than conventional elliptic polarized undulators. This is in particular important in SX beamlines in high-energy SR facilities, where a high *K* value is required to lower the fundamental photon energy. We have built a helical-8 undulator with a magnetic period of 120 mm and installed it in one of the SX beamlines in SPring-8 (BL17SU). Note that the total length is 3.6 m, and thus it also fits into the shorter straight section in SPring-8-II.

## 5. Summary

The concept of the greener upgrade of SPring-8 and the details of the system design are presented, which enables a significant improvement of the light source performance and a reduction of the power consumption of the entire facility. This upgrade also includes the integration of the two large accelerators, SPring-8 and SACLA, at the site, most of which has already been completed. The conventional dedicated injector has already been decommissioned, and the SACLA linear accelerator is operating as a time-shared injector for the SPring-8 storage ring. The prototypes of several key components for power savings, such as permanent-magnet-type bending magnets and DC septum magnets, have been developed. New undulators, IVU-II and helical-8, were developed and some of them have already been installed in the SPring-8 ring. For the ultralow emittance ring, the beam stability is critically important for fully exploiting its advantage. For this purpose, a transparent off-axis beam injection system and an optical-axis monitoring system are being developed. The experience and know-how gained from the XFEL user operation at SACLA will be very useful for the ultra-high-stability operation of SPring-8-II.

## Figures and Tables

**Table 1:** Main machine parameters for SPring-8-II and SPring-8

Parameter	SPring-8-II (new)	SPring-8 (present)
Lattice type	Five-bend	Double-bend
Energy (GeV)	6	8
Circumference (m)	1435.428	1435.949
Stored current (mA)	200	100
Emittance (pm rad)	50 with DWs (111 for bare lattice)	2400
Betatron function (m) at ID straight	8.2 / 2.8	31.2 / 5.0
Dispersion function (m) at ID straight	0.0	0.146
Tune ν_*x*_ / ν_*y*_	108.10 / 42.58	41.14 / 19.35
Natural chromaticity ξ_*x*_ / ξ_*y*_	−153 / −151	−117 / −47
Momentum compaction factor	4.13 × 10^−5^	1.60 × 10^−4^
Relative energy spread (%)	0.098	0.109
Damping partition number *J*_*x*_ / *J*_*y*_ / *J*_*s*_	1.38 / 1.0 / 1.62	1.0 / 1.0 / 2.0
Radiation loss by dipoles (MeV per turn)	2.6	8.9
Radiation loss by damping wigglers (MeV per turn per long-straight)	0.5	

**Table 2:** Main specifications of the lattice magnets

	LGB	NB	DQ	Q	S
Number of magnets in a cell	4	1	4	16	10
Maximum field/gradient	0.62 T	0.95 T	0.26 T, 23.8 T m^−1^	56 T m^−1^	3000 T m^−2^
Effective length	1550 mm	380 mm	350 mm	200–650 mm	100–300 mm
Good field region	≥ 6 mm	≥ 6 mm	≥ 6 mm	≥ 7 mm	≥ 7 mm
Gap/bore diameter	25 mm	25 mm	22 mm	34 mm	42 mm

**Table 3:** Main specifications of correction magnets

	O	Steering	Steering (in S)†	Steering (in O)†	Skew-Q (in O)†
Number of magnets in a cell	4	2	6	2	2
Maximum kick/gradient	100000 T m^−3^	0.12 mrad	0.15–0.45 mrad	0.20 mrad	0.29 T m^−1^
Effective length	150 mm	220 mm	100–300 mm	150 mm	150 mm

† Most steering and skew quadrupole functions are combined in the sextupole and the octupole magnets (see text).

**Table 4:** Alignment tolerances for the lattice magnets

	*x*, *y*	Yaw, pitch, roll
On-girder alignment	±0.05 mm	±0.1 mrad
Girder-to-girder alignment	±0.09 mm	±0.1 mrad

**Table 5:** Parameters of magnets relevant to beam injection

Parameter†	DC septum 1	DC septum 2	Pulsed septum	Bump kickers
Magnetic field (T)	1.4	1.4	1.4	0.2
Kick angle (mrad)	28	84	35	3

† Parameters are subject to change with future design revisions.
